# Two Antenna-Enriched Odorant Binding Proteins in *Dioryctria abietella* Tuned to General Odorants and Insecticides

**DOI:** 10.3390/insects13121145

**Published:** 2022-12-12

**Authors:** Chun Wu, Ningna Yin, Yuruo Guo, Zhengquan Wang, Naiyong Liu

**Affiliations:** Key Laboratory of Forest Disaster Warning and Control of Yunnan Province, Southwest Forestry University, Kunming 650224, China

**Keywords:** *Dioryctria abietella*, odorant binding protein, reproduction, ligand-binding assay, plant odorant, insecticide resistance, molecular simulation

## Abstract

**Simple Summary:**

*Dioryctria abietella* (Lepidoptera: Pyralidae) is a destructive forest pest that feeds on the shoots and cones of Pinaceae plants. The reception of host volatiles and toxic compounds can be mediated via odorant binding proteins (OBPs) expressed in olfactory organs. Here, a number of DabiOBPs may be involved in reproduction, as indicated by the expression profiles. Further, DabiOBP5 and DabiOBP14 enriched in adult antennae could strongly interact with two plant volatiles and four insecticides. Of these, β-ionone derived from plant flowers possibly served as an feeding attractant of female moths, while a pear ester ethyl-(2*E*,4*Z*)-decadienoate identified as a non-host volatile may repel *D. abietella*. In addition, two DabiOBPs were possibly associated with insecticide resistance. Molecular simulations identified several key residues involved in ligand-binding, revealing different binding mechanisms of DabiOBP5 and/or DabiOBP14 with the best ligands. These findings provide potentially active compounds for the control of *D. abietella* and characterize the interaction mechanisms of two antennal DabiOBPs with plant odorants and insecticides.

**Abstract:**

The management of forest pests has become a significant challenge, particularly for wood borers, because they spend most of the time in the trunks or cones. The coneworm, *Dioryctria abietella*, is a representative of cone borers as its larvae feed on the cones of Pinaceae plants. The molecular mechanisms underlying the interactions between this species and host plants or habitats can assist in developing strategies for pest control. In this study, we extended the expression profiles of 32 odorant binding proteins (OBPs) in the reproductive tissues of *D. abietella*, revealing the detectable transcription of 29 genes. Using two DabiOBPs highly expressed in antennae (DabiOBP5 and DabiOBP14) as targets, six compounds with high affinities (dissociation constants < 13 μM) were identified through a reverse chemical ecology strategy, including insecticides widely used for the control of lepidopteran pests. Of these compounds, a floral volatile β-ionone and a pear-produced ester ethyl-(2*E*,4*Z*)-decadienoate may serve as behaviorally active compounds in *D. abietella*. The strong binding of DabiOBPs to insecticides suggested their involvement in insecticide resistance, reflecting sophisticated detoxification mechanisms of this moth. In the molecular simulations, DabiOBP14 possessed stronger interactions with the six ligands compared to DabiOBP5, in which a few key residues within the binding pockets were involved in the formation of hydrogen bonds. This study provides some valuable reference active compounds for the development of lures or repellents in *D. abietella* and unravels the putative roles of two antenna-dominant DabiOBPs in the perception of plant-derived odorants and insecticides.

## 1. Introduction

The genus *Dioryctria* (Lepidoptera: Pyralidae) is composed of at least 79 described species, belonging to 11 species groups, including 15 representatives of five species groups distributed in China. As the larvae of the *Dioryctria* species mainly feed on the cones and shoots of the Pinaceae family, coupled with the concealed feeding habits of larvae and strong flight abilities of adults as well as the height of host plants, controlling these pests proves a challenge [1,2]. Many attempts to monitor and manage the pests have been made, including a pheromone-based environmentally-friendly strategy. In *Dioryctria abietella*, sex pheromones produced by female moths [(3*Z*,6*Z*,9*Z*,12*Z*,15*Z*)-pentacosapentaene and (9*Z*,11*E*)-tetradecadienyl acetate] were used for population monitoring and for the management of this pest in fields by designing strategies of pheromone-baited traps and mating distribution [3,4]. In addition, this pheromone-based control strategy was also applied to other *Dioryctria* species [5,6,7].

The odorant binding proteins (OBPs) of the Lepidoptera are of particular importance in guiding olfactory-related behaviors, and are therefore regarded as key molecular candidates for discriminating various odorant molecules, including sex pheromones and insecticides. In *Athetis lepigone*, a member of the antennal-binding protein X (ABP X) subfamily, AlepOBP6 highly expressed in adult antennae played crucial roles in the reception of sex pheromones and host volatiles, as is experimentally documented in the binding assays, molecular simulations and site-directed mutagenesis [8]. More recently, this integrated approach was also employed to unravel the putative roles of two antenna-dominant general OBPs (PxutGOBP1 and PxutGOBP2) in *Papilio xuthus*, suggesting their key involvement in the detection of host odorants and insecticides [9]. As indicated in other lepidopteran species, using antenna-enriched OBPs as target proteins to identify behaviorally active compounds has become an efficient strategy for the development and application of lures and repellents, coupled with further electrophysiological and behavioral assays [10,11]. Moreover, *in vivo* functional studies in moths have demonstrated specific correlations between OBPs and odorants. In *Cnaphalocrocis medinalis*, CmedOBP14 was able to detect two host volatiles, nerolidol and β-ionone, as evidenced by combining binding assays, RNA interference, electrophysiological and behavioral assays [12]. By using such an approach, SlitOBP7 and SlitGOBP2 in *Spodoptera litura* were suggested to mediate feeding behaviors of larvae [13].

The coneworm, *D. abietella*, is a severe forest pest, the larvae of which feeds on the cone and shoot tissues of Pinaceae plants. During the breeding season, egg-laying female adults search and locate suitable host plants, in which the young cones are their favorable oviposition sites [2,3]. In order to find the hosts, *D. abietella* utilize a main olfactory organ (i.e., antennae) to perceive and recognize host-produced volatiles, such as the terpene compounds released by *Picea likiangensis* var. *linzhiensis* [14]. Although the chemical communication between intraspecific (sex pheromone-mediating behaviors) and interspecific (plant volatile-mediating behaviors) interactions has been extensively studied, its molecular mechanisms remain largely unexplored, particularly for the identification of putative molecular targets in response to behaviorally active compounds. Prior to this study, DabiGOBP2 in *D. abietella* was shown to strongly interact with an attractant (dodecanal) and a repellent (myrcene) [15]. Outside of this DabiGOBP2, no other OBPs in this species have been functionally characterized, to date.

Our previous study identified 42 OBPs from the transcriptome of *D. abietella*, in which 37 of them were expressed in the antennae [16]. Here, we extended the expression characteristics of 32 OBPs in eight reproductive tissues of both sexes from *D. abietella*. Further, two antenna-enriched OBPs in this species, DabiOBP5 and DabiOBP14, were addressed through a combination of quantitative real-time PCR (qRT–PCR), binding assays and molecular docking simulations. These findings complement the existing information regarding the putative roles of OBPs in *D. abietella* and allow for the identification of more potentially active compounds used for the control of this pest.

## 2. Materials and Methods

### 2.1. Insect Rearing and Tissue Collection

The larvae and pupae of *D. abietella* were obtained from cones of *Pinus armandii* at Zixi Mountain, Chuxiong City, Yunnan Province, China. The collected cones were kept in nylon nets until the larvae developed into pupae. After pupation, female and male moths were distinguished by sex and were placed, separately, in different cages. The insects were reared following the conditions described by a previous study [16].

In order to determine the expression profiles of the DabiOBPs in the reproductive tissues of *D. abietella*, we excised and isolated reproductive systems of 3-day-old female and male moths, respectively. Phosphate-buffered saline (PBS, pH 7.4) was used to clean the fat bodies. The reproductive tissues were composed of accessory glands, ejaculatory ducts, seminal vesicles and testes for males, as well as accessory glands, bursa copulatrix, ovaries and spermathecae connecting spermathecal glands for females. In addition, 14 other adult tissues were also collected, representing antennae, heads without antennae, thoraxes, abdomens, legs and wings of both sexes, as well as female pheromone glands with ovipositors and male hairpencils. For each tissue, three biological pools were prepared.

### 2.2. Total RNA Extraction and Synthesis of First-Strand cDNA

We used TRIzol Regent (Ambion, Life Technologies, Carlsbad, CA, USA) to isolate the total RNA from each tissue, according to the manufacturer’s protocols. The genomic DNA was digested with gDNA Eraser, at 42 °C for 2 min, followed by the synthesis of first-strand cDNA using a PrimeScript RT Reagent Kit (TaKaRa, Dalian, China), under the conditions of 37 °C for 15 min and 85 °C for 5 s. In the expression profiling analyses of DabiOBPs, 5– and 15–fold dilutions of cDNA templates were used for reverse transcription (RT)–PCR and qRT–PCR assays, respectively.

### 2.3. Expression Profiling Analysis of DabiOBPs

In our previous study, 32 out of 42 DabiOBPs were studied in terms of their expression in 16 tissues of female and male adults via RT–PCR [16]. In order to examine the expression characteristics of these genes in reproductive tissues and to explore their putative roles in reproduction, we determined their transcription in eight reproductive-related tissues of both sexes. A TaKaRa Taq™ Kit (TaKaRa, Dalian, China) was applied to PCR amplification, with the primers listed in the previous study [16]. The reaction was performed with an annealing temperature of 58 °C and 35 cycles. The quality and usability of the templates were measured using a control gene, ribosomal protein L10 (DabiRPL10) [16]. Agarose gel electrophoresis was used to detect the absence and presence of OBP genes in the tissues.

Based on the results previously presented by RT–PCR and RNA sequencing (RNA–Seq), we selected two DabiOBPs in *D. abietella* that were highly expressed in the antennae to examine their relative expression levels in different tissues using qRT–PCR assays. Further, qRT–PCR was run on a qTOWER 2.2 instrument (Analytik Jena AG, Jena, Germany), using Bestar^®^ SybrGreen qPCR Mastermix (DBI Bioscience, Germany) and the primers designed by Beacon Designer 8.14 (PREMIER Biosoft International, Palo Alto, CA, USA) (Table S1). The qRT–PCR reaction was conducted at 95 °C for 2 min, followed by 40 cycles of 95 °C for 10 s, 58 °C for 31 s and 72 °C for 30 s. Three biological samples for each tissue and three technical replicates for each sample were conducted. The relative expression levels of the target OBP genes were normalized, relative to a control gene, DabiRPL8 [16], using a Q–Gene package [17,18].

In order to compare the expression differences of the DabiOBPs among the tissues, statistical analyses were conducted by one–way ANOVA followed by a Fisher’s least significant difference (LSD) test, implemented in IBM SPSS Statistics 21.0 (SPSS Inc., Chicago, IL, USA). When the *p* value was less than 0.05, the data were considered statistically significant.

### 2.4. Expression and Purification of Two Recombinant DabiOBPs

The nucleotide sequences of DabiOBP5 and DabiOBP14 were retrieved from the previously sequenced transcriptome [16]. First, signal peptides at N–termini of the proteins were removed based on the prediction by SignalP–6.0 [19]. Gene-specific primers of two DabiOBPs were designed, and restriction enzyme sites of forward (BamH I) and reverse (Xho I) primers were introduced with protective bases (Table S1). A high-fidelity DNA polymerase, PrimeSTAR^®^ Max DNA Polymerase (TaKaRa, Dalian, China), was used to clone the genes, using female antennal cDNA as templates. The amplification products were purified using a HiPure Gel Pure DNA Mini Kit (Magen, Guangzhou, China). The PCR products were digested with BamH I and Xho I, and were then ligated into an expression vector, pET–30a (+) previously digested by the above same enzymes, using a DNA Ligation Kit Ver.2.1 (TaKaRa, Dalian, China). The constructs, pET–30a (+)/DabiOBP5 and pET–30a (+)/DabiOBP14, were transformed into *Escherichia coli* DH5α competent cells, respectively. After being incubated overnight at 37 °C, positive clones were sequenced to confirm the correctness of the insertion sequences. The plasmids were harvested using a E.Z.N.A.^®^ Plasmid Mini Kit (Omega Bio-tek, Norcross, GA, USA).

In order to express the recombinant proteins, the plasmids were transformed into *E. coli* BL21 (DE3) competent cells. The expression and purification of recombinant pET–30a (+)/DabiOBPs were conducted, following the previously described procedures [20,21]. In short, a final concentration of 0.5 mM isopropyl β-D-thiogalactoside (IPTG) was used to induce the expression of proteins. The crude proteins were predominantly presented in the inclusion bodies and were denaturalized in the urea. The proteins were purified using an affinity column filled with Ni Sepharose 6FF (Solarbio Life Science, Beijing, China). The purified proteins were first dialyzed against buffers with a gradient of urea (6, 4, 3, 2, 1 and 0 M) so that their structures were renatured. The His-tag of the purified recombinant proteins was removed with treatments by recombinant enterokinase (GenScript, Nanjing, China).

### 2.5. Binding Assay

In the binding assays, we selected 100 compounds derived from host/non-host plants and man-made insecticides widely used for the control of lepidopteran pests, representing 88 general odorants (21 esters, 21 alcohols, 14 aldehydes, 13 alkenes, seven ketones, 11 alkanes and one acid) and 12 insecticides. These compounds and *N*-phenyl-1-naphthylamine (1-NPN) were purchased from Aladdin and Sigma-Aldrich, with the highest available purity. They were first diluted into 100 mM, using methanol (HPLC purity grade, ≥99.9%) as a stock solution. The concentrations of the work solution for each ligand and 1-NPN were set at 1 mM.

In order to measure the binding properties of the DabiOBPs to the 100 ligands, competitive binding assays were performed on a RF5301PC fluorescence spectrophotometer (Shimadzu, Japan). The concentrations of DabiOBPs and the fluorescent probe 1-NPN were set at 2 μM. The binding of DabiOBPs to 1-NPN in 20 mM Tris-HCl buffer (pH 7.4) was measured by adding final concentrations of 2–16 μM 1-NPN. Accordingly, binding constants (K_1-NPN_) of the DabiOBPs and 1-NPN were calculated using GraphPad Prism 7 (GraphPad Software Inc., San Diego, CA). The binding of the DabiOBPs to various ligands was conducted by adding different concentrations of ligands (2, 4, 6, 8, 12, 16 and 20 μM) into the DabiOBP5/1-NPN (each 2 μM) or DabiOBP14/1-NPN (each 2 μM) mixtures. For the ligands replacing over a half of 1-NPN fluorescence (50%) at 20 μM, three replicates were performed. The excitation wavelength was set at 337 nm and the emission peaks were recorded at around 400 nm. The dissociation constants (*K_i_*) of the tested ligands to DabiOBPs were computed, using the equation: *K_i_* = [IC_50_]/(1 + [1-NPN]/K_1-NPN_), where [1-NPN] was the free concentration of 1-NPN and IC_50_ was the concentration of ligands replacing 50% of 1-NPN fluorescence.

### 2.6. Homology Modeling and Molecular Docking

The homology templates of DabiOBP5 and DabiOBP14 were searched against the National Center for Biotechnology Information (NCBI) Protein Data Bank (PDB) database, using Position-Specific Iterated BLAST (https://blast.ncbi.nlm.nih.gov/Blast.cgi?PROGRAM=blastp&PAGE_TYPE=BlastSearch&LINK_LOC=blasthome, accessed on 20 July 2022). As a result, *Chrysopa pallens* OBP4 (CpalOBP4) [22] and *Anopheles gambiae* OBP4 (AgamOBP4) [23] shared the highest identities with DabiOBP5 and DabiOBP14, respectively. Therefore, their crystal structures (CpalOBP4, PDB ID: 6JPM; AgamOBP4, PDB ID: 3Q8I) were downloaded and modified, including the removal of water and ligands. Three-dimensional (3D) models of two DabiOBPs were generated with MODELLER 9*v*7 [24], using the CpalOBP4 and AgamOBP4 structures as templates. In the molecular docking simulations, we selected the common ligands of two DabiOBPs with high affinities (*K_i_* < 13 μM) to compare and analyze protein-ligand interactions and binding differences, i.e., ethyl-(2*E*,4*Z*)-decadienoate, β-ionone, chlorfenapyr, chlorpyrifos, phoxim and rotenone. The 3D structures of the chemicals were obtained from the NCBI PubChem database (https://pubchem.ncbi.nlm.nih.gov/, accessed on 12 July 2022). The protein and ligand structures were minimized using a CHARMm-based forcefield [25]. The chemicals were docked into the binding pockets of the DabiOBPs using Autodock Vina *v*1.1.2 [26]. The structures and docking results were visualized and edited using PyMOL *v*1.7.2.1 (https://pymol.org/2/, accessed on 20 July 2022).

## 3. Results

### 3.1. Identification of Candidate DabiOBPs Associated with Reproduction

**Figure 1:**
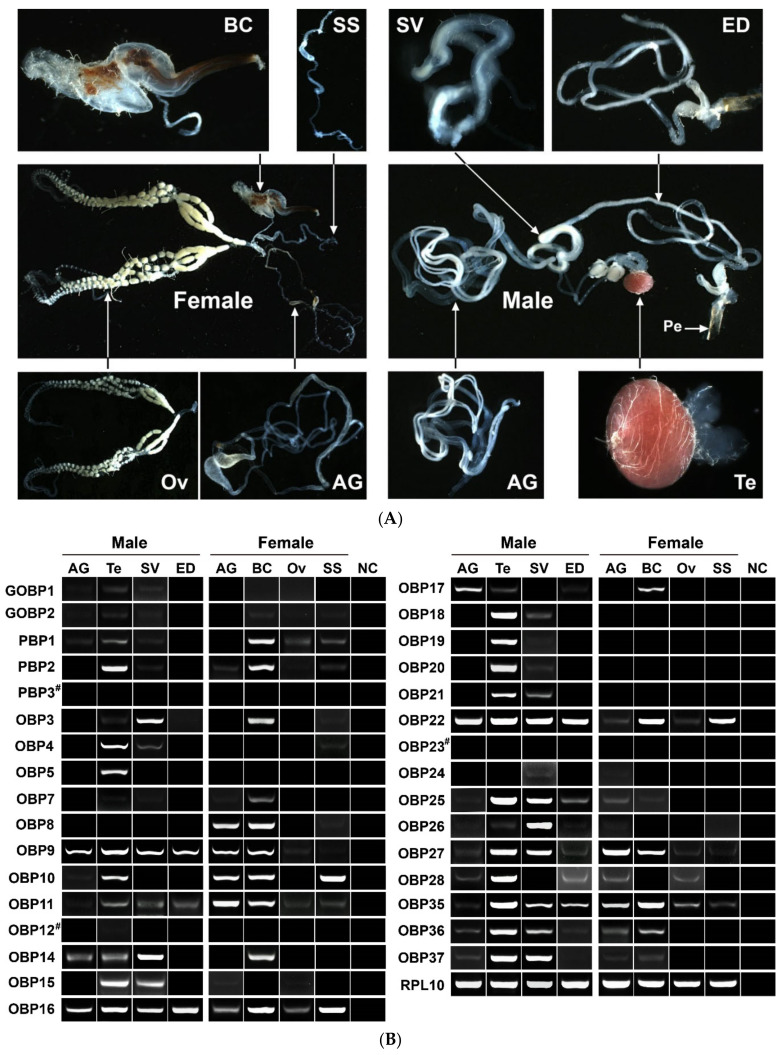
Expression profiles of DabiOBPs in eight reproductive tissues of *D. abietella* adults. (**A**) Reproductive systems of female and male adults. (**B**) Expression profiling analyses of 32 DabiOBPs in reproductive tissues. A control gene, DabiRPL10, was used to check the quality and integrity of the templates. AG, accessory glands; BC, bursa copulatrix; Ov, ovaries; SS, spermathecae with spermathecal glands; ED, ejaculatory ducts; SV, seminal vesicles and Te, testes. NC, negative control using sterile water as the template. # means that the transcription of genes was not detected in reproductive tissues. The original gel images are given in Supplementary Materials File S1.

Prior to this study, we identified 42 genes encoding OBPs in *D. abietella*, with 32 of them being characterized for their tissue expression profiles in various tissues of adults [16]. Focusing on the reproductive roles of DabiOBPs, here, the expression of the 32 genes was detected in reproductive-related tissues. The reproductive system of female *D. abietella* was composed of four parts, which included two accessory glands, one bursa copulatrix, eight oviducts and one spermatheca with a spermathecal gland. The reproductive system of the male moth comprised a pair of accessory glands, one ejaculatory duct, one testis and a pair of seminal vesicles (Figure 1A). In the RT–PCR analyses, 29 out of 32 were detected in one or more reproductive tissues, with the exception of DabiPBP3, OBP12 and OBP23. In comparison, four genes were found to have extremely low expression in all reproductive tissues, including DabiGOBP1, GOBP2, OBP7 and OBP24. Three DabiOBPs (DabiOBP16, OBP22 and OBP35) were broadly expressed in eight tissues. Of the three DabiPBPs, DabiPBP1 appeared to be enriched in bursa copulatrix, and DabiPBP2 were mainly presented in bursa copulatrix and testes. The majority of genes had detectable expression in the testes, representing 26 of 29 OBPs. Of the 26 testis-expressed OBPs, the expression of DabiOBP5 and DabiOBP19 was restricted to this tissue. In addition, we also detected a comparable number of OBPs in the seminal vesicles and bursa copulatrix, with 18 and 16 relatives, respectively (Figure 1B).

**Figure 2:**
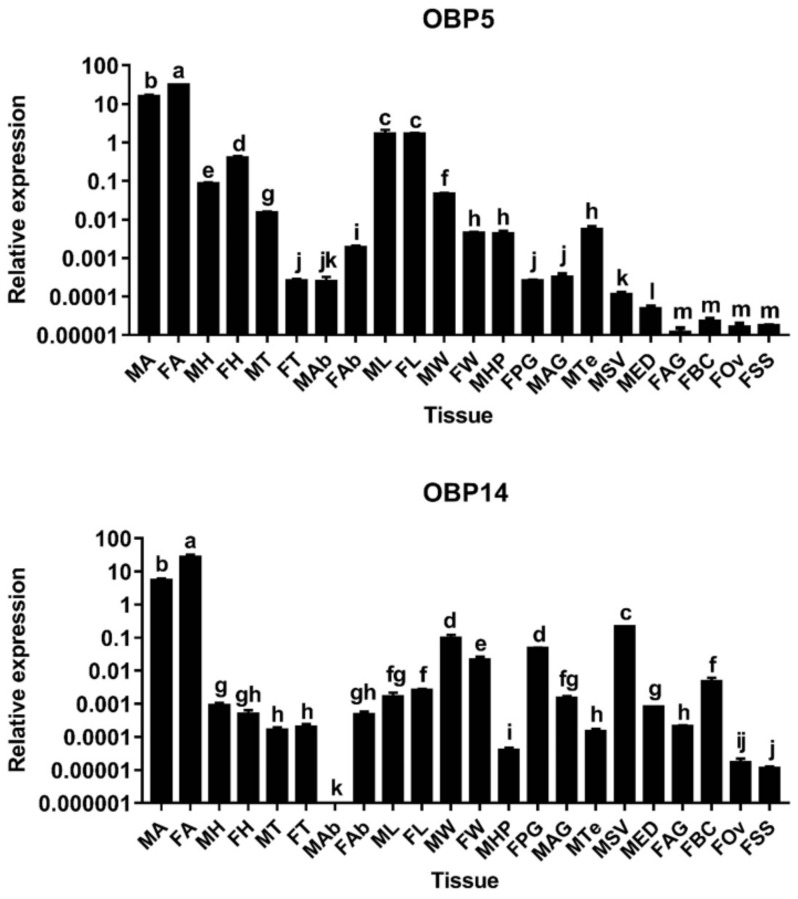
Relative expression of two candidate DabiOBP genes in various tissues of *D. abietella*. DabiRPL8 was used as a control gene to normalize the expression of target genes. MA, male antennae; FA, female antennae; MH, male heads without antennae; FH, female heads without antennae; MT, male thoraxes; FT, female thoraxes; MAb, male abdomens; FAb, female abdomens; ML, male legs; FL, female legs; MW, male wings; FW, female wings; MHP, male hairpencils; FPG, female pheromone glands; MAG, male accessory glands; MTe, male testes; MSV, male seminal vesicles; MED, male ejaculatory ducts; FAG, female accessory glands; FBC, female bursa coplatrix; FOv, female ovaries and FSS, female spermathecae with spermathecal glands. Different lowercase letters above the bars denote significant differences in gene expression levels among various tissues (ANOVA, LSD, *p* < 0.05). Error bars represent standard errors of three biological replicates.

Based on the FPKM (fragments per kilobase of transcript sequence per millions base pairs sequenced) and RT–PCR results obtained in both prior and current studies [16], we further examined the relative expression of two OBP genes (DabiOBP5 and DabiOBP14 showing high expression in antennae) by qRT–PCR. As expected, both of these genes were significantly expressed in the antennae of both sexes, with a female-biased level. In the female antennae, DabiOBP5 and DabiOBP14 displayed 1.98– and 4.86–fold higher expression compared to males, respectively. Although the expression of the two genes was also detected in most non-antennal tissues, their levels were extremely low (DabiOBP5: 9.22–fold differences between male antennae and male/female legs; DabiOBP14: 25.55–fold differences between male antennae and male seminal vesicles). Among eight of the reproductive tissues, DabiOBP5 had the highest expression in male testes, which supports the RT–PCR results of this gene. Similarly, the RT–PCR and qRT–PCR results of DabiOBP14 enriched in male seminal vesicles were identical (Figure 2).

### 3.2. Expression and Purification of DabiOBP5 and DabiOBP14

**Figure 3:**
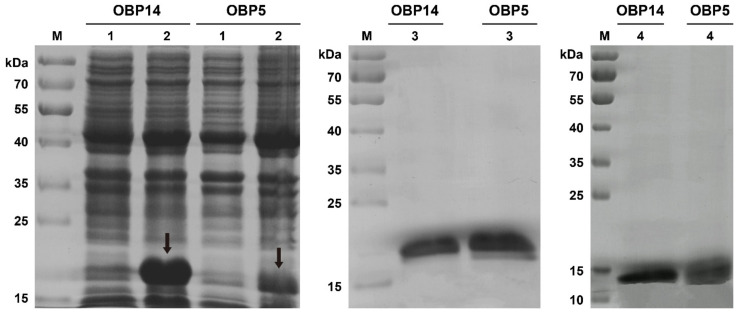
Expression and purification of two DabiOBPs in *D. abietella*, as indicated by SDS–PAGE. The crude bacterial extracts before (lane 1) and after (lane 2) IPTG induction, respectively. The purified recombinant proteins pET–30a (+)/DabiOBPs with the His-tag (lane 3). Re-purification of DabiOBPs after the removal of the His-tag (lane 4). M: Protein molecular weight marker. Arrows indicate induced target proteins pET–30a (+)/DabiOBPs.

Using a prokaryotic expression system, two recombinant pET–30a (+)/DabiOBPs were successfully induced and expressed with the expected sizes of bands (DabiOBP5: 19.54 kDa; DabiOBP14: 19.70 kDa). In comparison, pET–30a (+)/DabiOBP14 showed a good yield after IPTG induction. As the proteins were primarily presented in the inclusion bodies, denaturation and renaturation were performed in the urea. As a result, target proteins with a high purity were harvested. To avoid the effects of the His-tag on the ligand-binding assays of the DabiOBPs, the recombinant enterokinase was used to remove the His-tag. Finally, we obtained the target bands of DabiOBP5 and DabiOBP14 at 14.11 kDa and 14.27 kDa, respectively (Figure 3).

### 3.3. Binding Property of DabiOBP5 and DabiOBP14 to Ligands

**Figure 4:**
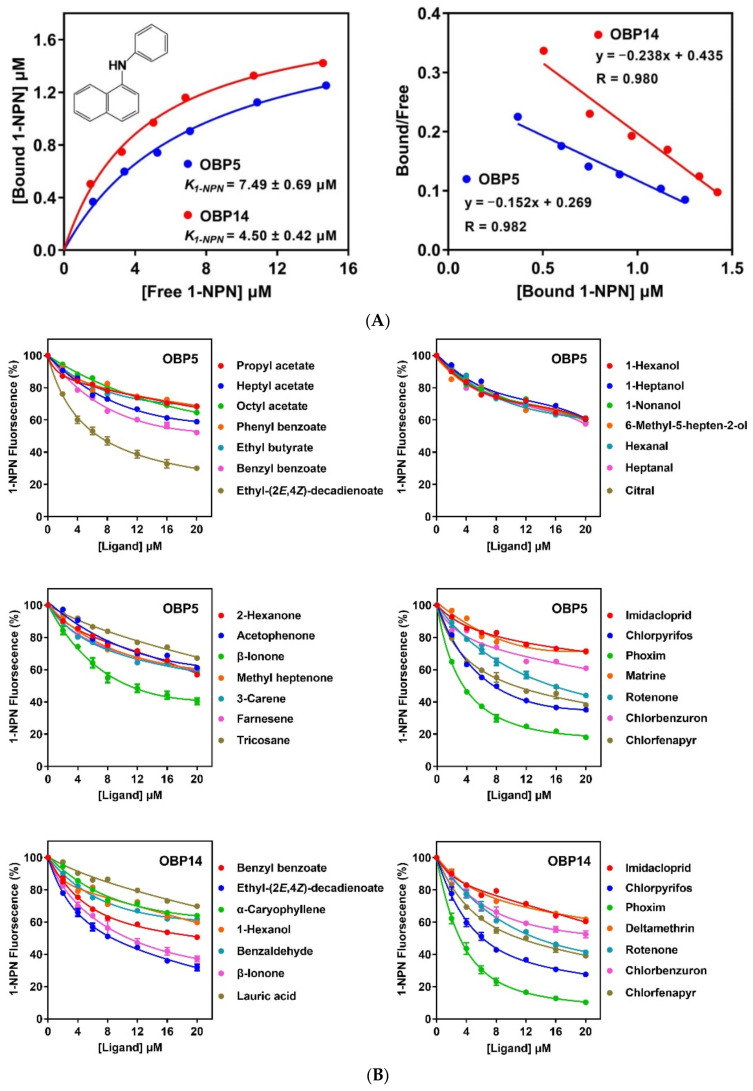
Binding property of selected ligands to two DabiOBPs in *D. abietella*. (**A**) Binding of two DabiOBPs to 1-NPN and relative Scatchard plots. (**B**) Competitive binding curves of two DabiOBPs to some ligands. For the ligands replacing more than 50% of 1-NPN fluorescence, three replicates were conducted. Each data point represents mean ± SE.

To evaluate whether the fluorescent reporter, 1-NPN, was able to bind with the two DabiOBPs, we monitored the changes of the fluorescent intensity and the peak of emission spectra. Both DabiOBP5 and DabiOBP14 could strongly interact with 1-NPN, with K_1-NPN_ values of 7.49 ± 0.69 μM and 4.50 ± 0.42 μM, respectively. The binding of DabiOBPs and 1-NPN was linearized, showing a good linear relationship (DabiOBP5: R = 0.982 and DabiOBP14: R = 0.980) (Figure 4A).

In order to identify the best ligands of DabiOBPs and their putative roles in chemosensation, a total of 100 chemicals were tested, representing the general odorants produced by host and non-host plants, as well as the insecticides used widely in the fields. Two DabiOBPs exhibited similar odorant binding spectra, including six common ligands with high affinities (*K_i_* < 13 μM). Of the six ligands, the *K_i_* values of five of the relatives were below 10 μM, with phoxim having the strongest interactions to DabiOBP5 (*K_i_* = 2.89 ± 0.14 μM) and DabiOBP14 (*K_i_* = 4.62 ± 0.46 μM). Intriguingly, two DabiOBPs were capable of binding a plant-derived insecticide, rotenone, with relatively high affinities (DabiOBP5: *K_i_* = 12.66 ± 0.63 μM; DabiOBP14: *K_i_* = 10.15 ± 0.49 μM). Outside the above six chemicals, DabiOBP5 exhibited a moderate binding with over one half of the remaining ligands (55/94), ranging between 30.49 and 47.80% of 1-NPN fluorescence. The remaining 39 chemicals showed weak or no binding to DabiOBP5. On the other hand, approximately 47% of the 94 ligands (44/94) were moderately bound by DabiOBP14, with a variable fluorescence displacement percentage of 30.02–49.39%. Nine out of the other 50 ligands had no binding to DabiOBP14, representing four alkanes, three alkenes, one alcohol and one insecticide. Among the chemicals with different functional groups, two DabiOBPs did not show an obvious binding preference. For the linear ligands showing different lengths of carbon chains, the DabiOBPs did not exhibit the correlation of binding, including 11 alkanes [heptane (7C) to tricosane (23C)], 10 acetates [methyl acetate (3C) to decyl acetate (12C)], eight alcohols [1-hexanol (6C) to 1-hexadecanol (16C)], and seven aldehydes [hexanal (6C) to dodecanal (12C)] (Figure 4B and Table S2).

### 3.4. Binding of Two DabiOBPs to the Optimal Ligands Reveals the Differences of Protein-Ligand Interactions

**Figure 5:**
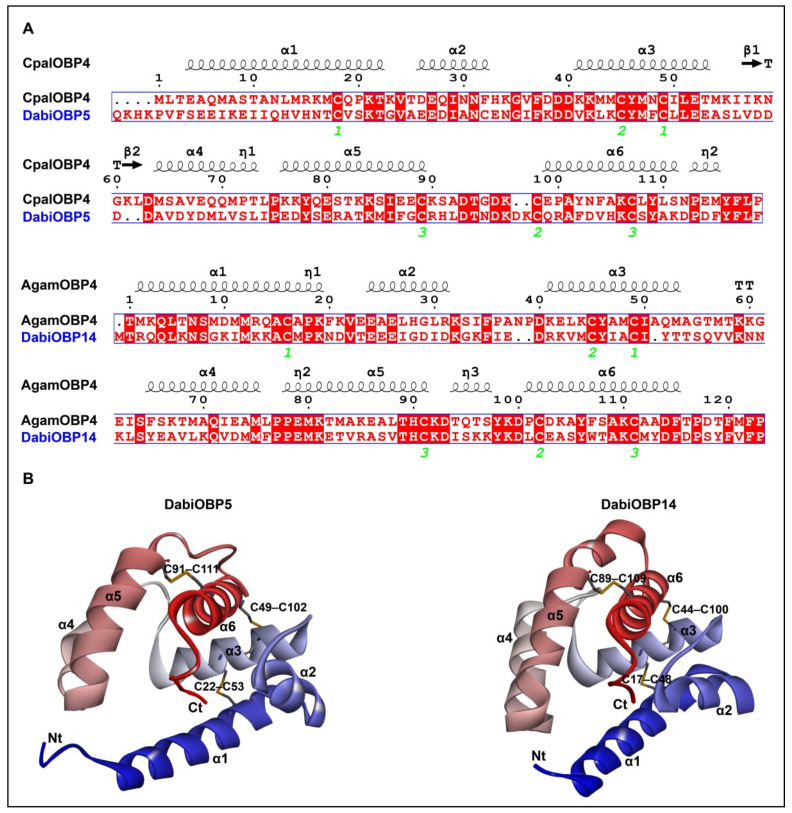
Three-dimensional (3D) structures of two DabiOBPs in *D. abietella*. (**A**) Alignments of amino acid sequences between DabiOBP5 and CpalOBP4 in *C. pallens*, or DabiOBP14 and AgamOBP4 in *A. gambiae*. Six α-helixes (α1–α6) are labeled on the top of the alignments. Green numbers 1 to 3 at the bottom of the alignments represent conserved cysteines forming three disulfide bonds. (**B**) Predicted 3D models of DabiOBP5 and DabiOBP14. N–terminus (Nt), C–terminus (Ct) and α-helixes are indicated, respectively.

To identify the key residues of the DabiOBPs in the ligand-binding and to compare their binding differences to the best ligands, the homology modeling and molecular docking of DabiOBP5 and DabiOBP14 were conducted. In the blast-based homology search of protein structures, CpalOBP4 from *C. pallens* and AgamOBP4 from *A. gambiae* shared the highest amino acid identities with DabiOBP5 (31.40%) and DabiOBP14 (39.02%) from *D. abietella*, respectively. Structural modeling revealed that 3D models of two DabiOBPs mainly consisted of six α-helices, representing α1 (residues 8–26), α2 (residues 30–36), α3 (residues 45–57), α4 (residues 66–75), α5 (residues 78–91) and α6 (residues 103–115) for DabiOBP5, as well as α1 (residues 3–20), α2 (residues 25–32), α3 (residues 40–51), α4 (residues 63–73), α5 (residues 76–89) and α6 (residues 99–113) for DabiOBP14. In comparison to CpalOBP4, DabiOBP5 possessed an extended N–terminus. Between CpalOBP4 and DabiOBP5, only the α3 helix shared over 50% identity at the amino acid levels, while the α4 helix had no identical amino acids. Between AgamOBP4 and DabiOBP14, both α1 and α5 helices exhibited 50% amino acid identities (Figure 5A). Based on the predicted 3D models, the binding pockets of two of the DabiOBPs were formed by five α-helices (α1, α3, α4, α5 and α6), two Loops (Loop 3 between α3 and α4, Loop5 between α5 and α6) and a C–terminal tail, respectively. The structures were stabilized by three disulfide bonds (DabiOBP5: C22–C53, C49–C102 and C91–C111; DabiOBP14: C17–C48, C44–C100 and C89–C109) (Figure 5B).

**Figure 6:**
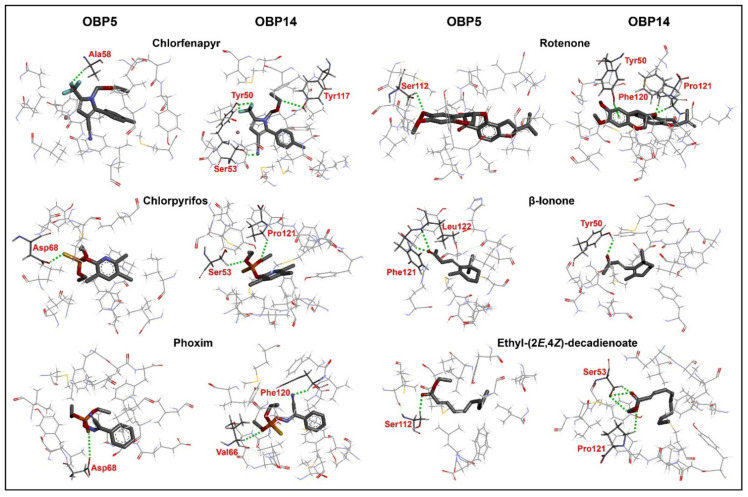
Molecular docking of DabiOBPs to six common ligands. Green dashed lines represent H–bonds between ligands and DabiOBPs. Residues forming inter-molecular H–bonds are shown in red. Detailed information on the interactions of ligands and proteins is listed in Table 1.

Using 3D models of the DabiOBPs as templates, we docked the best six ligands into their respective binding pockets. Two DabiOBPs exhibited different interaction mechanisms, including binding energies, driving forces and key residues. DabiOBP14 appeared to have stronger interactions than DabiOBP5 with a same ligand, as lower binding energies and more hydrogen bonds (H–bonds, except for β-ionone) were obtained in the ligand-binding of DabiOBP14. Moreover, a larger number of residues were involved in the interactions of DabiOBP14 and ligands relative to DabiOBP5. Focusing on a primary driving force (H–bonds) between the protein-ligand interactions, several key amino acids were identified in the binding pockets. For the molecular docking of DabiOBP5 and its ligands, β-ionone could strongly interact with the residues, in which two H–bonds were formed between this ligand and Phe121 or Leu122. The interactions of DabiOBP5 and the other five compounds were also involved in the formation of H–bonds, with one for each compound. For the interactions of DabiOBP14 and its ligands, several residues contributed to the generation of H–bonds, including Tyr50, Ser53, Phe120 and Pro121 (Table 1 and Figure 6).

## 4. Discussion

Insect OBPs have been documented to serve multiple functions, but, to date, they are still regarded as one of the main carriers of hydrophobic odorant molecules, particularly for those that are highly or specifically expressed in the antennae [27]. As a forest pest whose larvae feed on the cones and seeds of Pinaceae plants, the management of *D. abietella* has received increasing attention in recent years, particularly in Europe and China [3,28]. To efficiently control this pest, some attractants (including sex pheromones) and repellents have been identified and tested in the laboratory and in seed orchards [4,14]. In this paper, we have addressed the putative roles of two antenna-dominant DabiOBPs in *D. abietella* with respect to their expression characteristics and ligand-binding properties, revealing the interaction mechanisms of the OBP-ligand binding and providing some potentially active compounds for the design and development of lures or repellents.

In our prior study, a number of antenna- or proboscis-expressed OBPs were detected, suggestive of their roles in smell and taste [16]. Our current work extends the knowledge of the putative non-chemosensory roles of DabiOBPs in reproduction. As indicated by isolating the reproductive systems of female and male *D. abietella*, their constituents were similar to those in *S. litura*, *Achelura yunnanensis* and *P. xuthus*. Notably, in the three lepidopteran species, olfactory-related genes were found to have expression in the reproductive tissues, including OBPs, chemosensory proteins (CSPs) and odorant receptors (ORs) [9,29,30]. Furthermore, OBPs enriched in the male reproductive systems of *Helicoverpa armigera* and *S. litura* likely modulate female behaviors by transferring the proteins to females during copulation [30,31]. In *D. abietella*, a number of DabiOBPs were expressed in the testes and seminal vesicles of males, as well as in the female bursa copulatrix, all of which are associated with reproduction. For those OBPs specifically or highly presented in the male reproductive tissues of *D. abietella*, they were likely to serve as modulators of female behaviors. In addition, the OBPs in the male reproductive tissues also may contribute to diverse tasks, such as development and stress resistance, as demonstrated in the DmelOBP50a and DmelOBP50d of *Drosophila melanogaster*, respectively [32].

In *D. abietella*, 18 out of the 42 OBPs appeared to have abundant expression in the antennae, in which their FPKM values were higher than those in other tissues [16]. Unfortunately, the RT–PCR data of the DabiOBPs were unable to identify antenna-enriched or sex-biased genes, largely limiting the selection of target OBPs for subsequent functional studies. Based on the FPKM values of the DabiOBPs in various tissues, we selected two DabiOBPs that were highly expressed in the antennae (DabiOBP5: FPKM = 2398.71 in females and 1184.25 in males; DabiOBP14: FPKM = 1393.39 in females and 809.91 in males) to further measure their relative expression levels in the tissues. In line with the results of the RNA–Seq, both of the DabiOBPs were significantly enriched in the antennae at female-biased levels. In other insects, OBPs specifically or highly presented in the antennae have been indicated to be involved in host orientation and partner recognition [8,13,33]. In some case, sex-biased OBPs were capable of guiding female or male specific behaviors, such as CquiOBP1 in *Culex quinquefasciatus* [34], BodoOBP5 in *Bradysia odoriphaga* [35] and GfunGOBP3 in *Grapholita funebrana* [36] for female oviposition behaviors, as well as BodoOBP1 and BodoOBP2 in *B. odoriphaga* [37] and AlepOBP6 in *A. lepigone* [8] for male mating behaviors. Given the high and female-biased expression of two of the DabiOBPs in the principle olfactory organ, it was postulated that the two DabiOBPs were likely to sense odorants derived from host or non-host plants, with an emphasis on the putative roles in detecting oviposition-related compounds.

Unexpectedly, the two DabiOBPs did not strongly bind the terpene compounds generally produced by Pinaceae plants [14]. The majority of these compounds only had a moderate binding affinity with the two DabiOBPs, ranging between 30 and 40% in the fluorescent displacement percentage. This can most likely be explained by the hypothesis that DabiOBP5 and DabiOBP14 may detect non-terpene odorants, whereas the terpene compounds are most likely to be recognized by other OBPs from *D. abietella*, such as DabiGOBP2 [15]. Indeed, the two DabiOBPs were able to strongly interact with ethyl-(2*E*,4*Z*)-decadienoate (a pear-derived volatile) and β-ionone (an ordinary floral odorant). Of the two compounds, ethyl-(2*E*,4*Z*)-decadienoate exists mainly in ripe fruits and is an attractant for tortricid moths [38,39]. Herein, this compound is released by non-host plants of *D. abietella* and may play a role in repelling this moth. On the other hand, β-ionone is commonly emitted by flowers of plants [40]. In *D. abietella*, the strong binding activities of the two DabiOBPs to β-ionone were possibly involved in the feeding behaviors of adults by visiting nectar flowering plants. In fact, the flowering plants also need to employ animals, such as insects, to help their pollination. β-Ionone emitted by the flowers may be a key scent for attracting pollinating insects; this is further supported by the observation that soluble olfactory proteins in other species, referring to OBPs and CSPs enriched in antennae, could detect this compound [9,41,42,43,44,45].

Insecticide resistance has become a common issue for the chemical control of pests, particularly for insecticide residue which is threatening human and animal health. Insects, the largest group among animals, directly or indirectly touch the insecticides left on the surface of the leaves, branches or trunks of host and non-host plants. To adapt to the changing external environment, insects have evolved diverse detoxification systems, in which they employ some members (e.g., OBPs and CSPs) other than detoxification enzymes to improve their own resistance to insecticides [27,46,47]. Our current study found that four insecticides widely used for the control of lepidopteran pests could strongly interact with the two DabiOBPs, in which chlorpyrifos and phoxim were also detected by OBPs from other insects [48,49,50,51]. Adult *D. abietella* use the antennae to search and locate suitable host plants to suck and for oviposition. Furthermore, in some case, they have to stay on non-host plants due to a long-duration flight. In these life activities of *D. abietella*, some olfactory proteins presented in the antennae and/or tarsi (e.g., OBPs) may sense insecticides at a close distance or through a contact strategy in order to evaluate the fitness of the plants, as well as to improve the insecticide resistance of adults. Thus, the antenna- and leg-expressed characteristics of two DabiOBPs further highlight the above hypothesis, supporting their putative roles in the detection of insecticides.

## 5. Conclusions

This study extended the expression profiles of 32 DabiOBPs in the reproductive tissues of *D. abietella*, revealing 29 reproductive-related OBPs. The qRT–PCR analyses showed that DabiOBP5 and DabiOBP14 displayed significantly high expression in the adult antennae, suggesting their putative olfactory roles in host seeking and orientation. Using the two purified DabiOBPs as targets, it was suggested that they may be involved in the perception of host/non-host odorants, representing a floral volatile β-ionone and a pear ester ethyl-(2*E*,4*Z*)-decadienoate. On the other hand, two DabiOBPs possibly participated in insecticide resistance, i.e., they may improve the tolerance of this pest to toxic compounds by binding partial insecticides. Notably, two DabiOBPs possessed different ligand-binding interactions with the best compounds. These findings address the binding molecular mechanisms of antennal binding proteins and potentially active compounds in *D. abietella*, facilitating the development and application of lures and repellents for the control of this species.

## Figures and Tables

**Table 1:** Binding energies and key residues of DabiOBPs to the optimal ligands.

Chemical	Binding Energy (kcal/mol)	Residue Forming Intra-Molecular H–Bond	Residue Within 4 Å (Red Residues Forming Inter-Molecular H–Bond between DabiOBPs and Ligands)
OBP5			
Chlorfenapyr	–4.3	L55–L60	I11, I14, V18, A58, L60, D68, Y69, L72, I76, M87, F121, L122, F123
Chlorpyrifos	–4.1	–	I11, I14, L55, A58, L60, D68, Y69, L72, I76, M87, F121, L122, F123
Phoxim	–4.8	–	I11, I14, V18, L55, E57, A58, L60, D68, Y69, L72, I76, M87, F121, L122, F123
Rotenone	2.1	H109–S112	I11, I14, L55, A58, L60, Y69, L72, I76, M87, I88, V108, H109, S112, F121, L122, F123
β-Ionone	3.2	R83–S112, V108–S112, H109–S112	L60, Y69, R83, M87, I88, C91, V108, H109, S112, F121, L122, F123
Ethyl-(2*E*,4*Z*)-decadienoate	–2.9	V108–S112, H109–S112	A58, L60, Y69, L72, I76, M87, I88, C91, V108, S112, F121, L122, F123
OBP14			
Chlorfenapyr	–7.7	L6–G10	L6, K7, M13, Y50, S53, Q54, V66, Q69, M73, F74, V82, V86, M110, Y117, F118, V119, F120, P121
Chlorpyrifos	–5.8	V66–Q69, V66–V70	M13, I49, Y50, S53, V66, Q69, V70, M73, F74, V82, V86, M110, Y117, F118, V119, F120, P121
Phoxim	–5.3	L6–G10, Y50–Q54, V66–Q69, V66–V70	L6, S9, M13, Y50, S53, Q54, V66, Q69, V70, M73, F74, V82, V86, M110, F118, V119, F120, P121
Rotenone	–3.7	L6–G10, Y50–Q54	L6, K7, G10, M13, Y50, S53, Q54, V66, Q69, M73, F74, V86, M110, F118, V119, F120, P121
β-Ionone	–5.4	–	M13, Y50, S53, Q54, V66, Q69, F74, V82, M110, Y117, F118, V119, F120, P121
Ethyl-(2*E*,4*Z*)-decadienoate	–5.3	V66–Q69, V66–V70	M13, I49, Y50, S53, V66, Q69, V70, F74, V82, V86, M110, Y117, F118, V119, F120, P121

## Data Availability

All the data generated in this study can be found in the article and Supplementary Materials.

## Supplementary Materials

The following are available online at: https://www.mdpi.com/article/10.3390/insects13121145/s1, File S1: Uncropped gel pictures of candidate OBPs in *D. abietella*; Table S1: Primers used for the expression profiles and prokaryotic expression of DabiOBPs in *D. abietella*; Table S2: Binding affinities of two *D. abietella* OBPs to various ligands.
