# Supplementary material for: Two Antenna-Enriched Odorant Binding Proteins in Dioryctria abietella Tuned to General Odorants and Insecticides
Source: Insects. 2022 Dec 12;13(12):1145. doi: 10.3390/insects13121145 (PMC9781003; doi:10.3390/insects13121145)
Supplement: Supplementary file 1 [file insects-13-01145-s001.zip › File S1.pptx]

## Slide 1
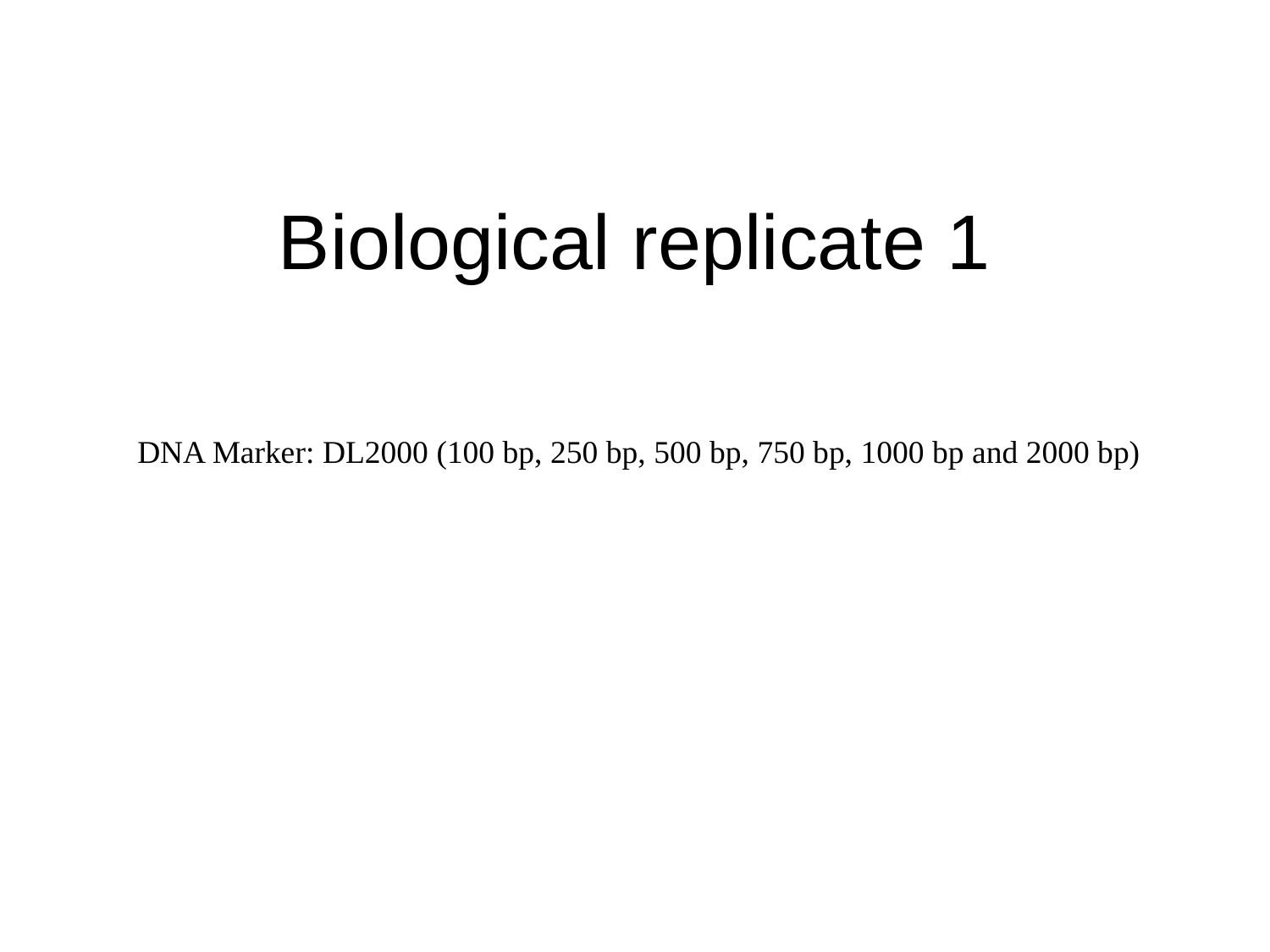

# Biological replicate 1
DNA Marker: DL2000 (100 bp, 250 bp, 500 bp, 750 bp, 1000 bp and 2000 bp)

## Slide 2
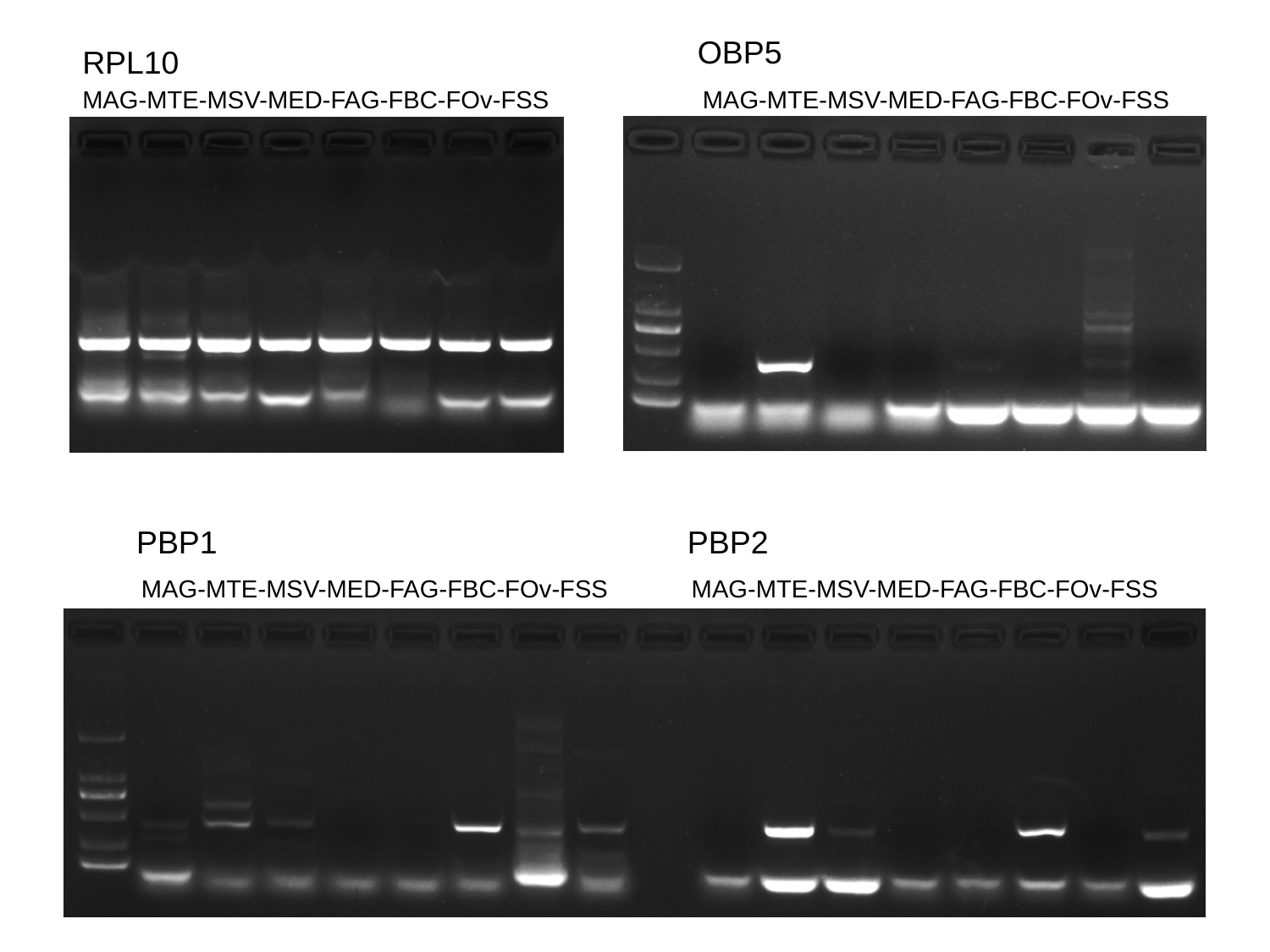

OBP5
RPL10
MAG-MTE-MSV-MED-FAG-FBC-FOv-FSS
MAG-MTE-MSV-MED-FAG-FBC-FOv-FSS
PBP1
PBP2
MAG-MTE-MSV-MED-FAG-FBC-FOv-FSS
MAG-MTE-MSV-MED-FAG-FBC-FOv-FSS

## Slide 3
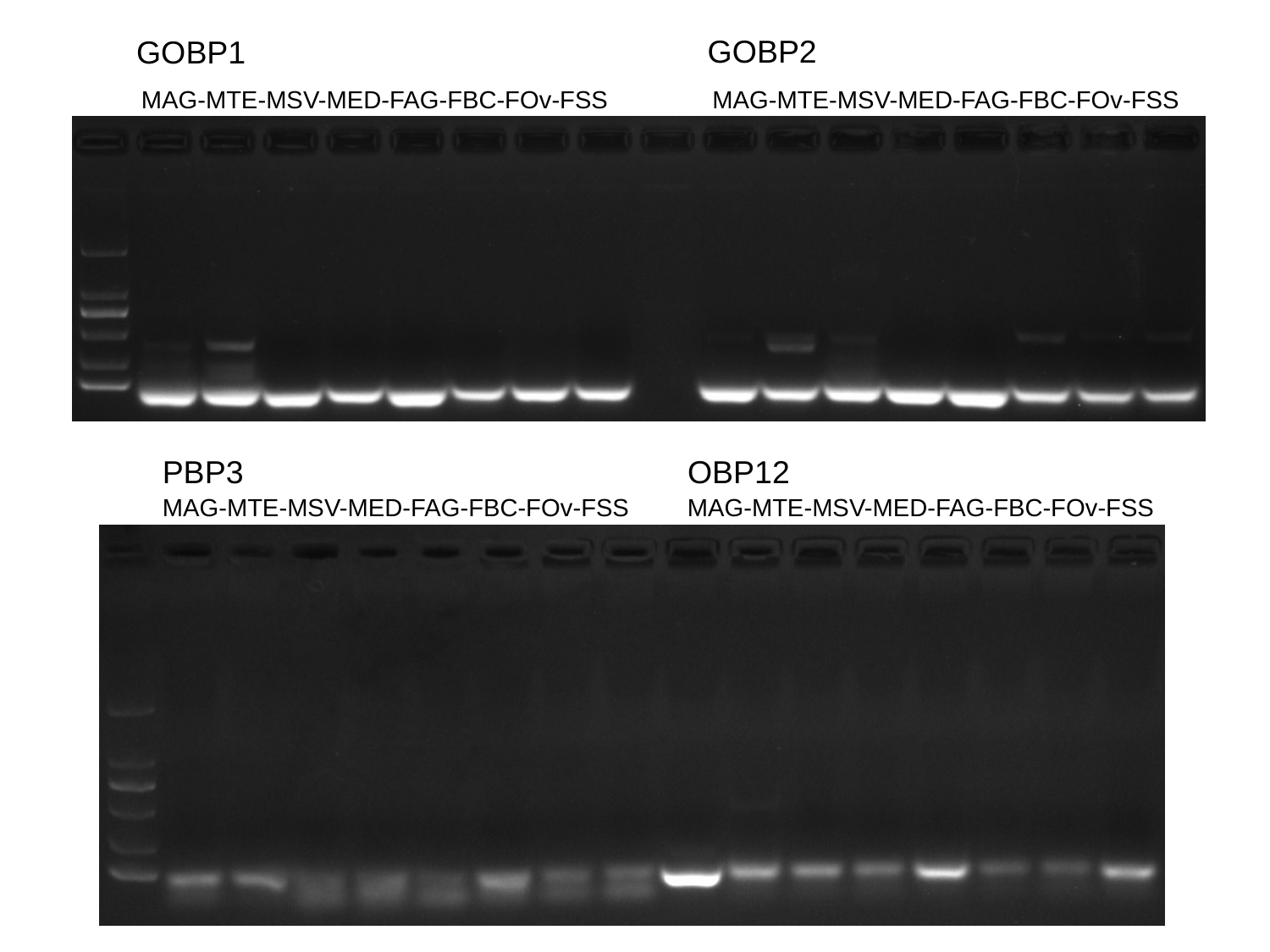

GOBP2
GOBP1
MAG-MTE-MSV-MED-FAG-FBC-FOv-FSS
MAG-MTE-MSV-MED-FAG-FBC-FOv-FSS
OBP12
PBP3
MAG-MTE-MSV-MED-FAG-FBC-FOv-FSS
MAG-MTE-MSV-MED-FAG-FBC-FOv-FSS

## Slide 4
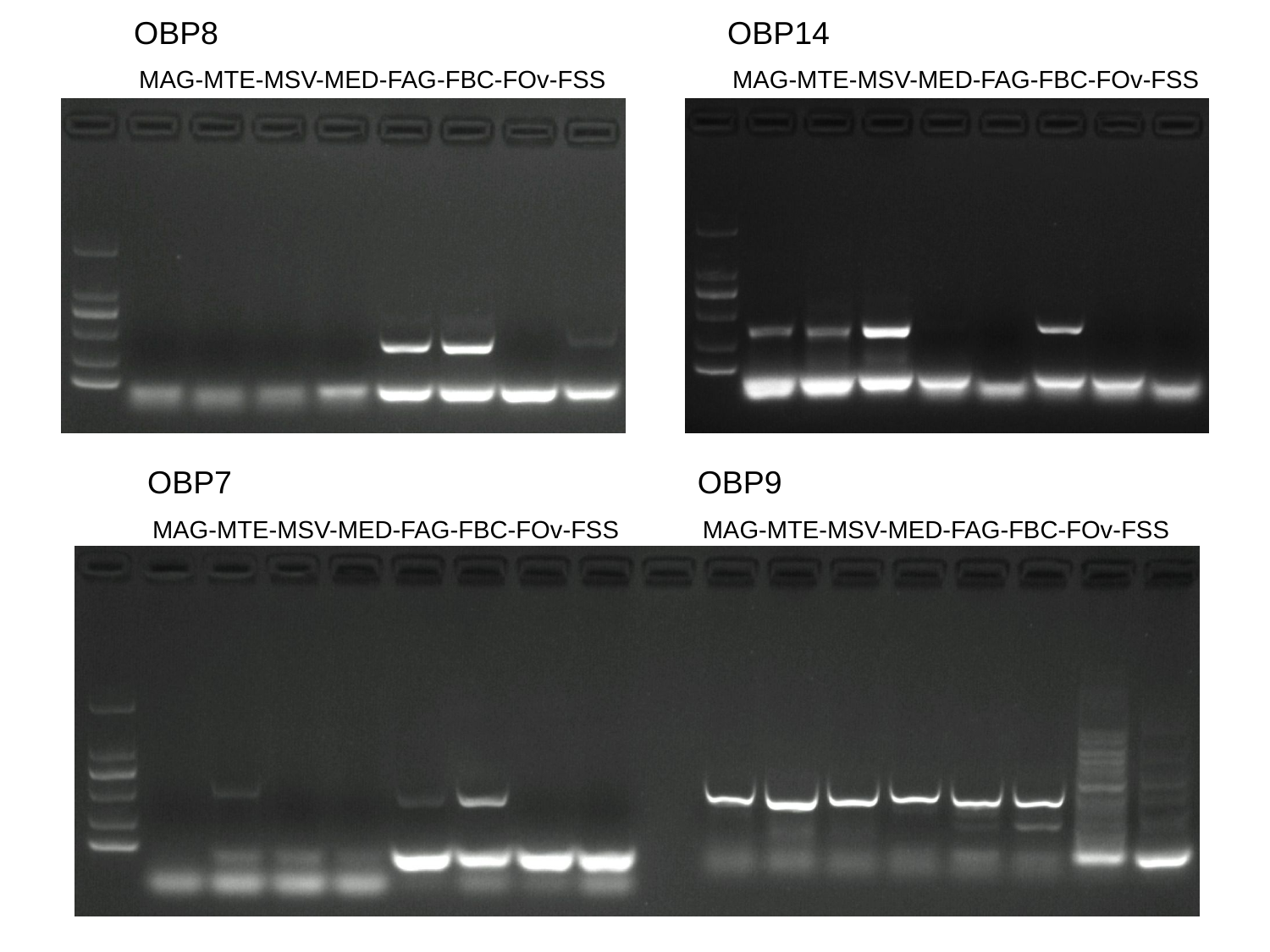

OBP8
OBP14
MAG-MTE-MSV-MED-FAG-FBC-FOv-FSS
MAG-MTE-MSV-MED-FAG-FBC-FOv-FSS
OBP7
OBP9
MAG-MTE-MSV-MED-FAG-FBC-FOv-FSS
MAG-MTE-MSV-MED-FAG-FBC-FOv-FSS

## Slide 5
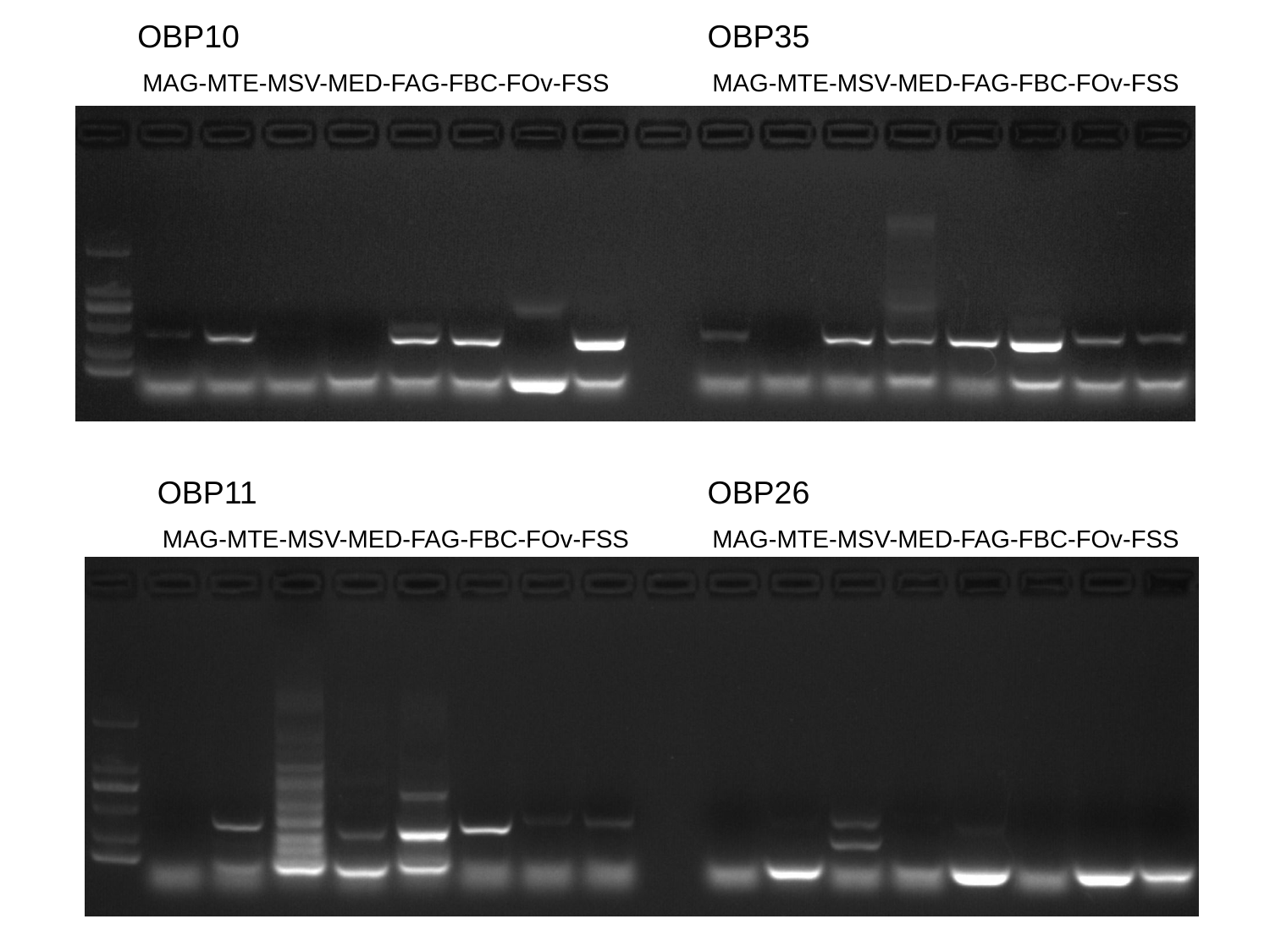

OBP10
OBP35
MAG-MTE-MSV-MED-FAG-FBC-FOv-FSS
MAG-MTE-MSV-MED-FAG-FBC-FOv-FSS
OBP11
OBP26
MAG-MTE-MSV-MED-FAG-FBC-FOv-FSS
MAG-MTE-MSV-MED-FAG-FBC-FOv-FSS

## Slide 6
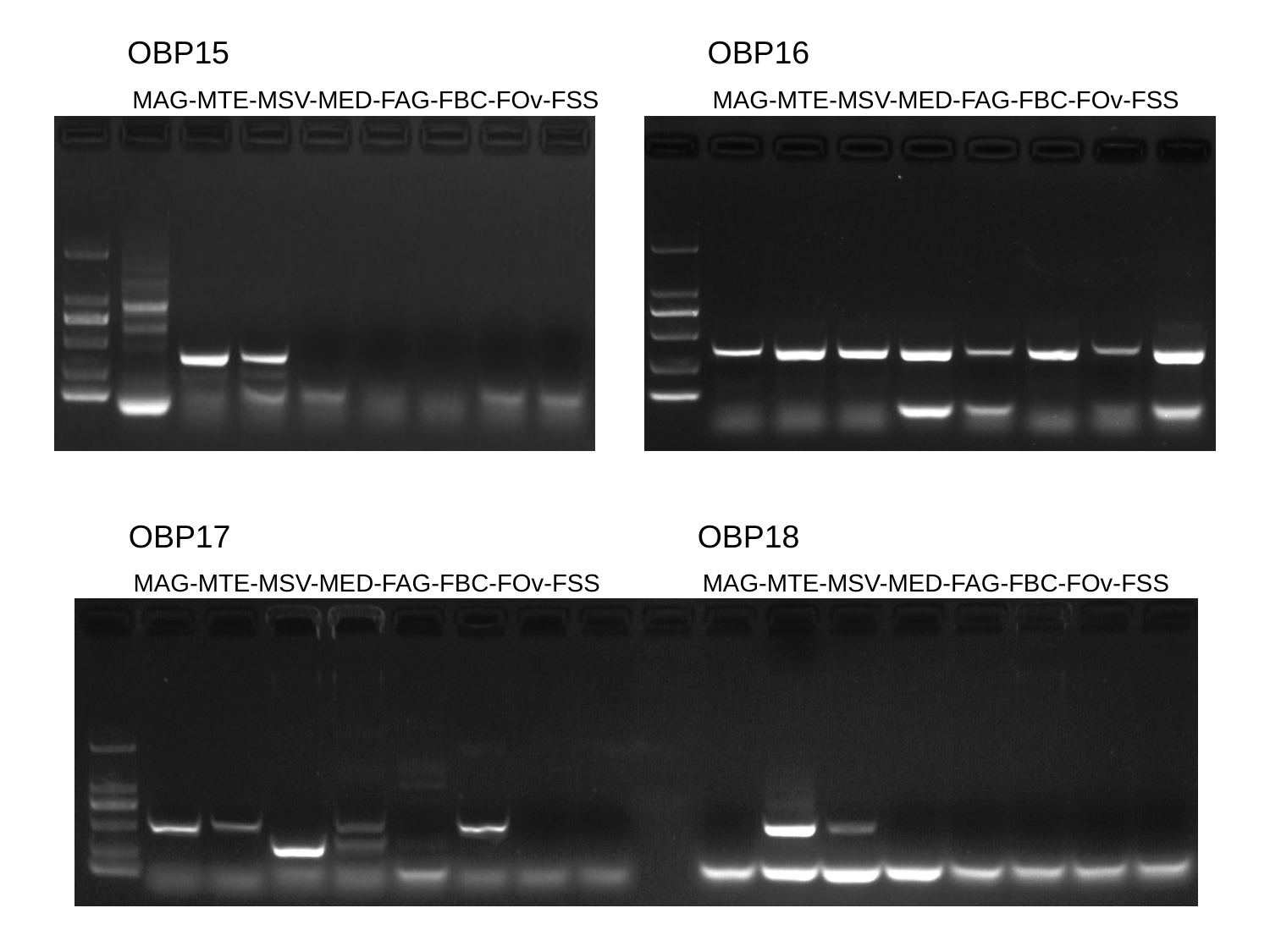

OBP15
OBP16
MAG-MTE-MSV-MED-FAG-FBC-FOv-FSS
MAG-MTE-MSV-MED-FAG-FBC-FOv-FSS
OBP17
OBP18
MAG-MTE-MSV-MED-FAG-FBC-FOv-FSS
MAG-MTE-MSV-MED-FAG-FBC-FOv-FSS

## Slide 7
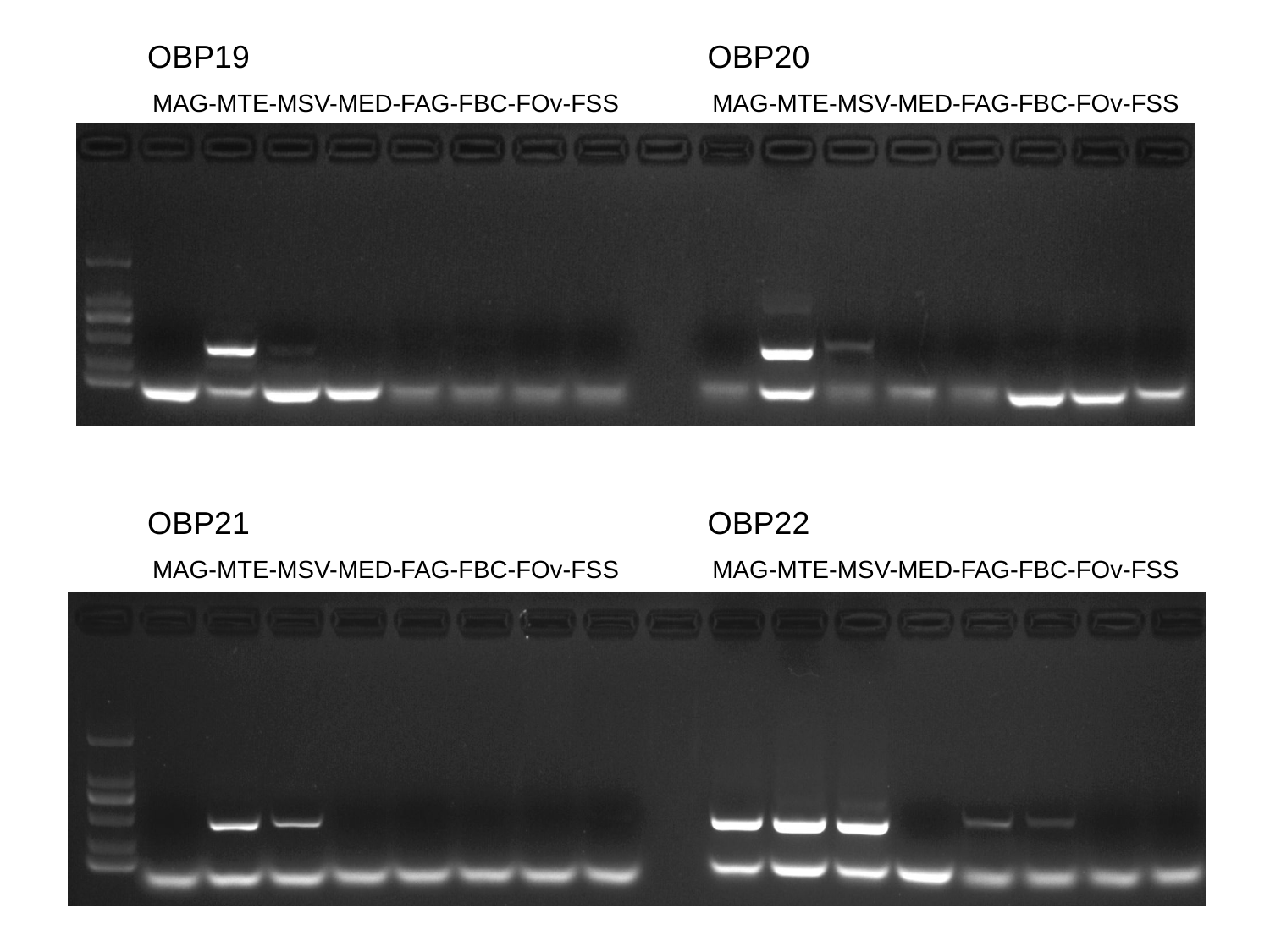

OBP19
OBP20
MAG-MTE-MSV-MED-FAG-FBC-FOv-FSS
MAG-MTE-MSV-MED-FAG-FBC-FOv-FSS
OBP21
OBP22
MAG-MTE-MSV-MED-FAG-FBC-FOv-FSS
MAG-MTE-MSV-MED-FAG-FBC-FOv-FSS

## Slide 8
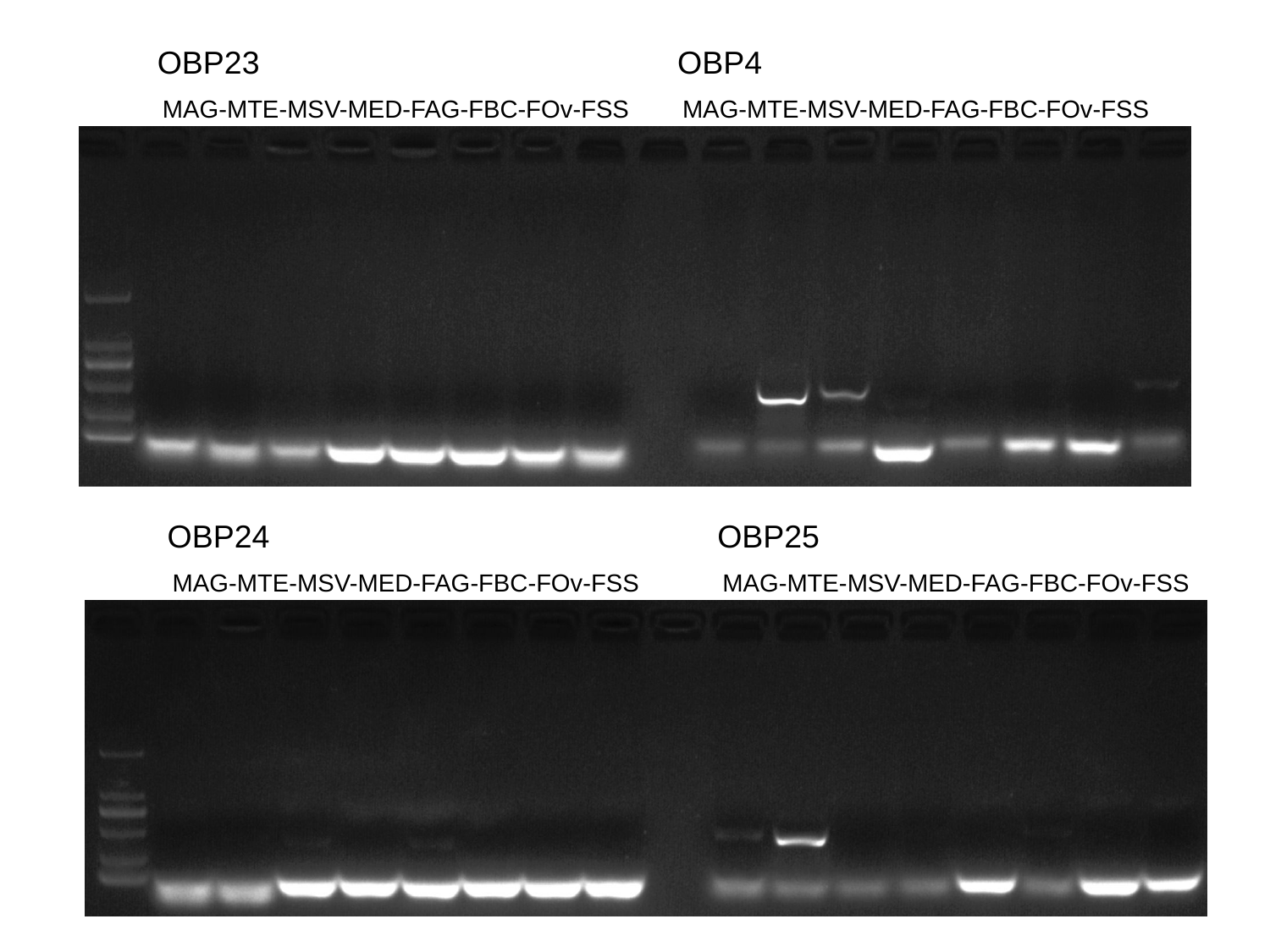

OBP23
OBP4
MAG-MTE-MSV-MED-FAG-FBC-FOv-FSS
MAG-MTE-MSV-MED-FAG-FBC-FOv-FSS
OBP24
OBP25
MAG-MTE-MSV-MED-FAG-FBC-FOv-FSS
MAG-MTE-MSV-MED-FAG-FBC-FOv-FSS

## Slide 9
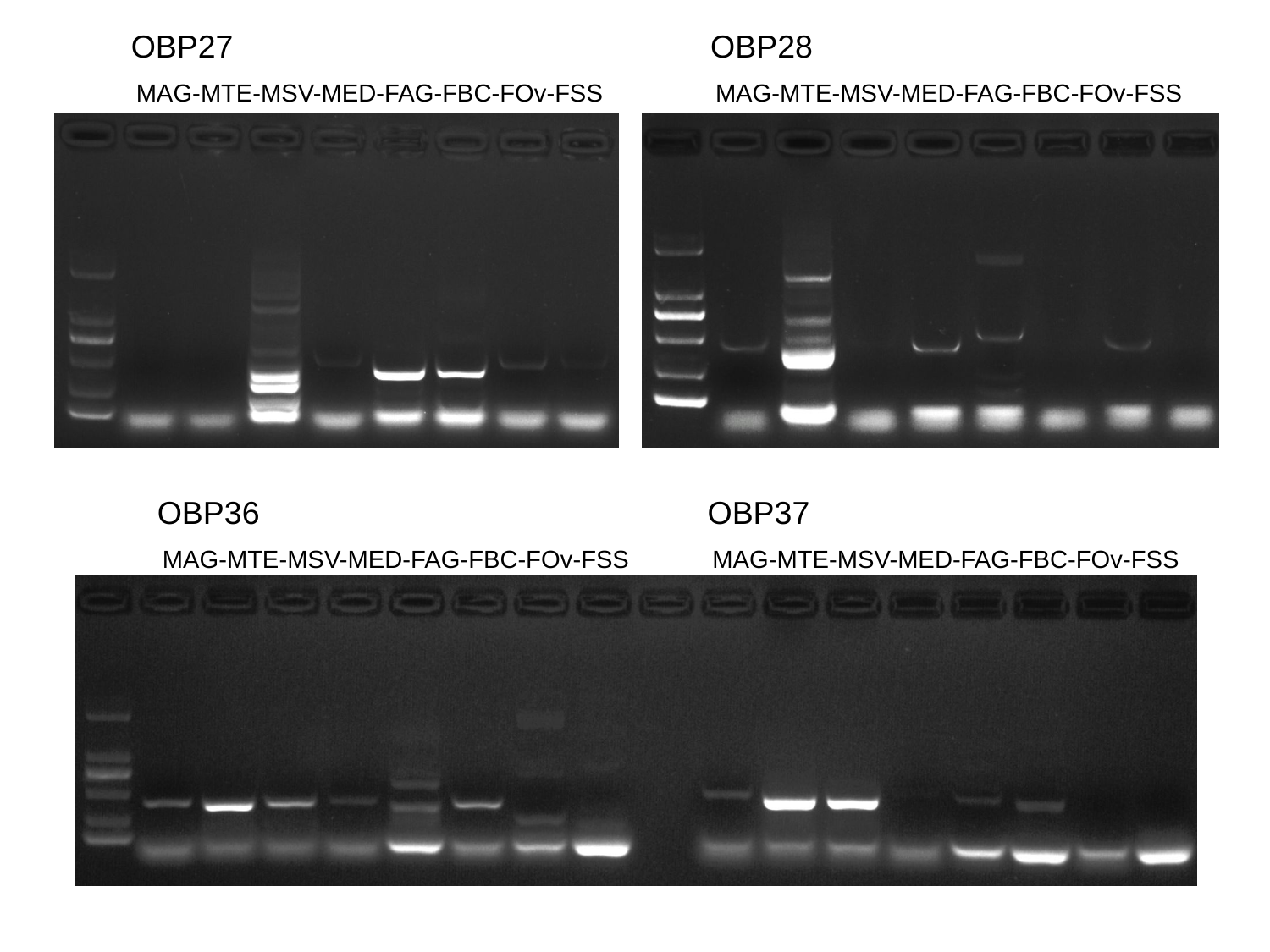

OBP27
OBP28
MAG-MTE-MSV-MED-FAG-FBC-FOv-FSS
MAG-MTE-MSV-MED-FAG-FBC-FOv-FSS
OBP36
OBP37
MAG-MTE-MSV-MED-FAG-FBC-FOv-FSS
MAG-MTE-MSV-MED-FAG-FBC-FOv-FSS

## Slide 10
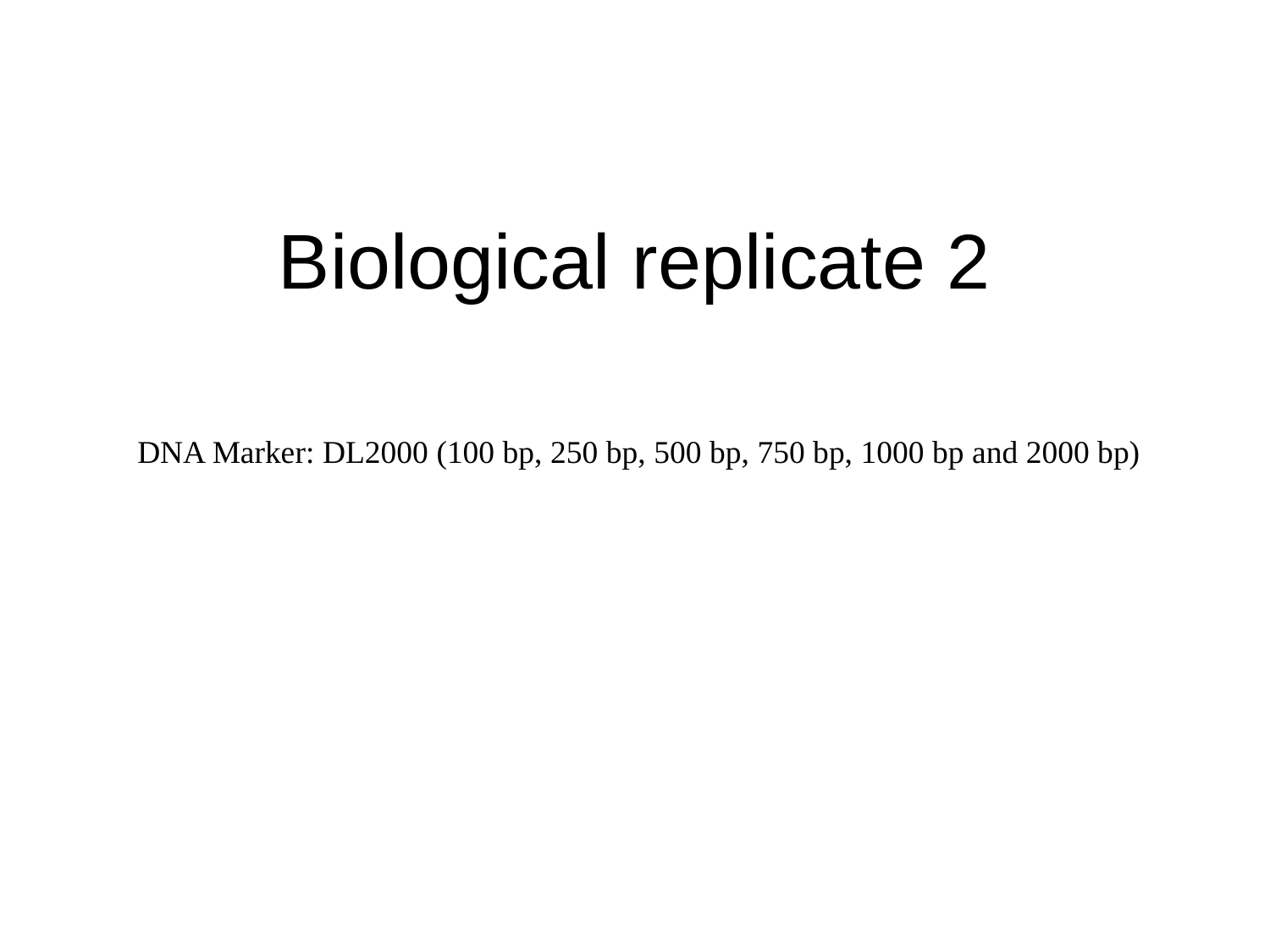

# Biological replicate 2
DNA Marker: DL2000 (100 bp, 250 bp, 500 bp, 750 bp, 1000 bp and 2000 bp)

## Slide 11
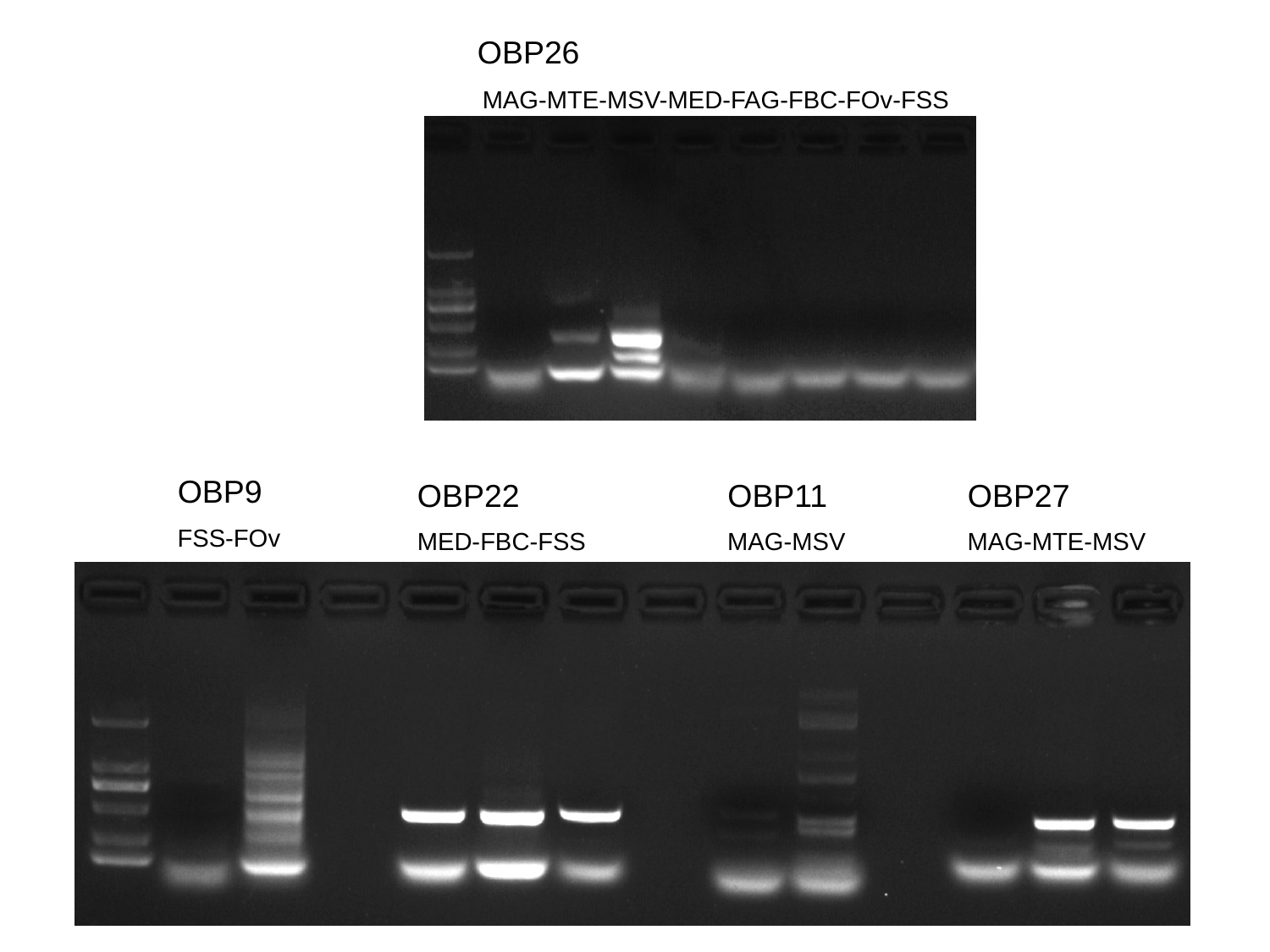

OBP26
MAG-MTE-MSV-MED-FAG-FBC-FOv-FSS
OBP9
OBP22
OBP11
OBP27
FSS-FOv
MED-FBC-FSS
MAG-MSV
MAG-MTE-MSV

## Slide 12
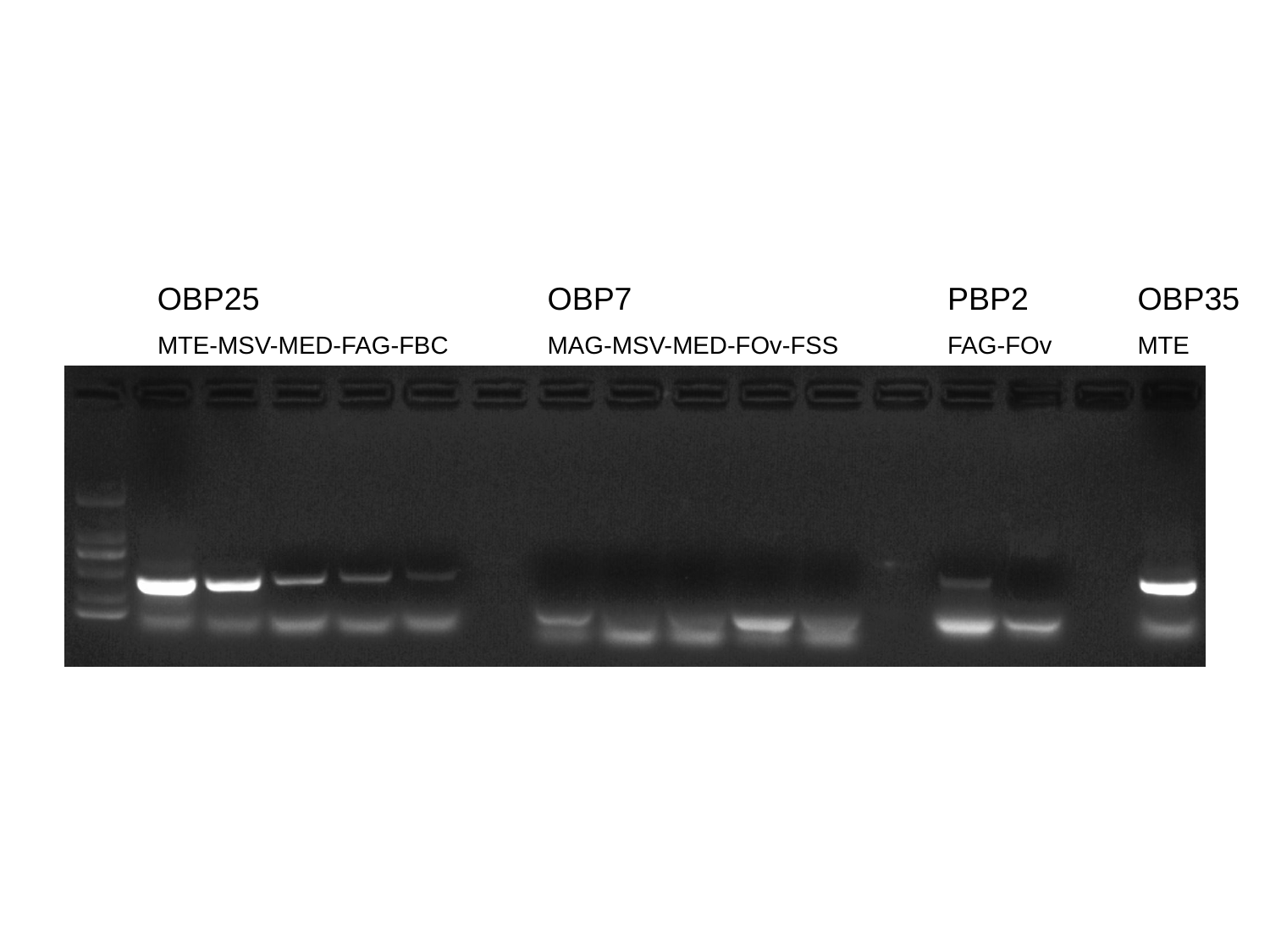

OBP25
OBP7
PBP2
OBP35
MTE-MSV-MED-FAG-FBC
MAG-MSV-MED-FOv-FSS
FAG-FOv
MTE
