# Supplementary material for: Two Antenna-Enriched Odorant Binding Proteins in Dioryctria abietella Tuned to General Odorants and Insecticides
Source: Insects. 2022 Dec 12;13(12):1145. doi: 10.3390/insects13121145 (PMC9781003; doi:10.3390/insects13121145)
Supplement: Supplementary file 1 [file insects-13-01145-s001.zip › Table S1.pdf]

Table S1. Primers used for the expression profiles and prokaryotic expression of DabiOBPs in *D. abietella*.

Gene	Primer sequence (5'–3')	
	**Forward primer**	**Reverse primer**
**qRT–PCR analysis**		
OBP5	AGGAGGATATAGCGAATTGC	CGGAGTAGTCTTCTGGTATC
OBP14	GCTTGTATGCCTAAGAACG	TCTGGAGGGAACATCATATC
RPL8	GGCATGGTTGGAATTGTT	AGTTACGCTTCACCTTGT
**Prokaryotic expression**		
OBP5	CGGGATCCCAAAAACATAAACCTGTTTTTAGTG	CCCTCGAGTTAGAATAAAAAGTAGAAATCAGGGT
OBP14	CGGGATCCATGACGCGACAGCAGTTGAA	CCCTCGAGTTATGGAAACACAAAATAGCTTGGA

Note: Restriction enzyme sites (forward: BamH I and reverse: Xho I) are underlined.
