# Supplementary material for: Two Antenna-Enriched Odorant Binding Proteins in Dioryctria abietella Tuned to General Odorants and Insecticides
Source: Insects. 2022 Dec 12;13(12):1145. doi: 10.3390/insects13121145 (PMC9781003; doi:10.3390/insects13121145)
Supplement: Supplementary file 1 [file insects-13-01145-s001.zip › Table S2.pdf]

Table S2. Binding affinities of two *D. abietella* OBPs to various ligands.

Ligand	CAS number	OBP5		OBP14	
		F.D. (%)	K_i (μM)	F.D. (%)	K_i (μM)
**Esters**					
Methyl acetate	79-20-9	27.62	–	27.21	–
Ethyl acetate	141-78-6	18.99	–	23.29	–
Propyl acetate	109-60-4	31.42	–	25.46	–
Butyl acetate	123-86-4	31.57	–	0.16	–
Amyl acetate	628-63-7	26.33	–	25.00	–
Hexyl acetate	142-92-7	23.71	–	29.22	–
Heptyl acetate	112-06-1	41.04	–	33.70	–
Octyl acetate	112-14-1	35.41	–	30.09	–
Nonyl acetate	143-13-5	16.47	–	22.98	–
Decyl acetate	112-17-4	26.24	–	15.15	–
Geranyl acetate	105-87-3	26.51	–	27.39	–
*Z*3-Hexenyl acetate	3681-71-8	27.72	–	31.25	–
*Z*3-Hexenyl butyrate	16491-36-4	20.80	–	26.39	–
Ethyl butyrate	105-54-4	31.98	–	31.72	–
Methyl benzoate	93-58-3	30.49	–	30.65	–
Ethyl benzoate	93-89-0	29.39	–	30.02	–
Phenyl benzoate	93-99-2	31.69	–	34.36	–
Benzyl benzoate	120-51-4	47.80	–	49.39	–
Ethyl-(2*E*,4*Z*)-decadienoate	3025-30-7	69.83	**5.67±0.55**	68.07	**6.45±0.43**
Ethyl hexanoate	123-66-0	24.17	–	29.43	–
Methyl laurate	111-82-0	7.25	–	12.60	–
**Alcohols**					
1-Hexanol	111-27-3	38.88	–	40.27	–
1-Heptanol	111-70-6	39.32	–	39.10	–
2-Heptanol	543-49-7	13.90	–	24.86	–
1-Octanol	111-87-5	34.97	–	35.51	–
1-Nonanol	143-08-8	39.58	–	29.98	–
1-Decanol	112-30-1	31.40	–	31.32	–
1-Undecanol	112-42-5	37.66	–	NB	–
1-Dodecanol	112-53-8	38.67	–	28.55	–
*Z*3-Hexen-1-ol	928-96-1	28.57	–	24.52	–
Linalool	78-70-6	33.33	–	31.71	–
Farnesol	4602-84-0	36.29	–	36.20	–
Geraniol	106-24-1	32.91	–	28.67	–
Nerolidol	7212-44-4	34.62	–	37.52	–
Phenethyl alcohol	60-12-8	37.61	–	10.00	–
2-Methyl-1-butanol	137-32-6	36.04	–	27.78	–
3-Methyl-2-buten-1-ol	556-82-1	36.95	–	34.58	–
1-Hexadecanol	36653-82-4	36.97	–	31.97	–

3-Octenol	3391-86-4	25.07	–	31.31	–
*E*2-Hexen-1-ol	928-95-0	36.50	–	18.64	–
α-Terpineol	98-55-5	33.89	–	30.28	–
6-Methyl-5-hepten-2-ol	4630-06-2	41.37	–	33.49	–
**Aldehydes**					
Hexanal	66-25-1	39.33	–	32.96	–
Heptanal	111-71-7	42.46	–	25.87	–
Octanal	124-13-0	35.56	–	34.48	–
Nonanal	124-19-6	31.50	–	23.55	–
Decanal	112-31-2	29.13	–	21.35	–
Undecanal	112-44-7	27.83	–	35.28	–
Dodecanal	112-54-9	30.61	–	3.43	–
*Z*2-Hexenal	16635-54-4	39.42	–	33.09	–
*E*2-Hexenal	6728-26-3	33.10	–	32.34	–
Syringaldehyde	134-96-3	38.53	–	34.32	–
β-Cyclocitral	432-25-7	32.90	–	32.77	–
Citral	5392-40-5	39.02	–	32.25	–
Benzaldehyde	100-52-7	36.78	–	38.35	–
Phenylacetaldehyde	122-78-1	29.15	–	33.05	–
**Alkenes**					
Ocimene	13877-91-3	33.97	–	33.18	–
α-Pinene	80-56-8	25.12	–	29.24	–
Farnesene	502-61-4	40.68	–	17.77	–
α-Caryophyllene	6753-98-6	15.42	–	NB	–
β-Caryophyllene	87-44-5	16.28	–	NB	–
1-Undecene	821-95-4	17.44	–	NB	–
Myrcene	123-35-3	37.07	–	34.84	–
Squalene	7683-64-9	32.30	–	24.81	–
3-Carene	13466-78-9	41.63	–	29.92	–
γ-Terpinene	99-85-4	28.23	–	24.46	–
α-Phellandrene	99-83-2	37.38	–	36.07	–
α-Terpinene	99-86-5	35.10	–	27.93	–
Limonene	138-86-3	38.31	–	30.81	–
**Ketones**					
2-Hexanone	591-78-6	43.11	–	27.57	–
3-Hexanone	589-38-8	25.30	–	36.58	–
2-Heptanone	110-43-0	36.47	–	33.53	–
2-Tridecanone	593-08-8	35.58	–	32.61	–
Acetophenone	98-86-2	38.75	–	36.22	–
β-Ionone	14901-07-6	59.51	**9.11±1.03**	62.86	**8.39±1.04**
6-Methyl-5-hepten-2-one	110-93-0	40.28	–	34.39	
**Alkanes**					
Heptane	142-82-5	31.42	–	22.91	–
Octane	111-65-9	27.95	–	NB	–

Nonane	111-84-2	22.19	–	NB	–
Decane	124-18-5	20.23	–	NB	–
Undecane	1120-21-4	4.42	–	6.47	–
Dodecane	112-40-3	31.90	–	17.71	–
Tridecane	629-50-5	5.52	–	NB	–
Tetradecane	629-59-4	25.00	–	27.13	–
Octadecane	593-45-3	26.53	–	23.94	–
Nonadecane	629-92-5	27.73	–	15.30	–
Tricosane	638-67-5	32.82	–	27.90	–
**Insecticides**					
Abamectin	71751-41-2	24.16	–	4.33	–
Imidacloprid	138261-41-3	22.51	–	39.56	–
Diflubenzuron	35367-38-5	21.40	–	30.33	–
Chlorbenzuron	57160-47-1	39.11	–	47.43	–
Deltamethrin	52918-63-5	28.53	–	38.21	–
Chlorpyrifos	2921-88-2	66.12	**5.92±0.48**	72.41	**4.62±0.46**
Phoxim	14816-18-3	81.98	**2.89±0.14**	89.62	**2.38±0.28**
Fipronil	120068-37-3	23.68	–	29.70	–
Chlorfenapyr	122453-73-0	61.98	**8.16±0.55**	60.84	**8.41±0.42**
Rotenone	83-79-4	56.04	**12.66±0.63**	58.66	**10.15±0.49**
Matrine	519-02-8	29.01	–	29.59	–
Osthole	484-12-8	NB	–	NB	–
**Acids**					
Lauric acid	143-07-7	33.87	–	30.18	–

Note: F.D. represents the fluorescence displacement percent of ligands at 20 μM. NB means no binding of DabiOBPs to ligands.
